# Defects of mitochondrial RNA turnover lead to the accumulation of double-stranded RNA *in vivo*

**DOI:** 10.1371/journal.pgen.1008240

**Published:** 2019-07-31

**Authors:** Aleksandra Pajak, Isabelle Laine, Paula Clemente, Najla El-Fissi, Florian A. Schober, Camilla Maffezzini, Javier Calvo-Garrido, Rolf Wibom, Roberta Filograna, Ashish Dhir, Anna Wedell, Christoph Freyer, Anna Wredenberg

**Affiliations:** 1 Department of Medical Biochemistry and Biophysics, Karolinska Institutet, Stockholm, Sweden; 2 Max Planck Institute Biology of Ageing - Karolinska Institutet Laboratory, Karolinska Institutet, Stockholm, Sweden; 3 Department of Molecular Medicine and Surgery, Karolinska Institutet, Stockholm, Sweden; 4 Centre for Inherited Metabolic Diseases, Karolinska University Hospital, Stockholm, Sweden; 5 Centre for Genomic and Experimental Medicine, MRC Institute of Genetics and Molecular Medicine, University of Edinburgh, Edinburgh, United Kingdom; University of Cologne, GERMANY

## Abstract

The RNA helicase SUV3 and the polynucleotide phosphorylase PNPase are involved in the degradation of mitochondrial mRNAs but their roles *in vivo* are not fully understood. Additionally, upstream processes, such as transcript maturation, have been linked to some of these factors, suggesting either dual roles or tightly interconnected mechanisms of mitochondrial RNA metabolism. To get a better understanding of the turn-over of mitochondrial RNAs *in vivo*, we manipulated the mitochondrial mRNA degrading complex in *Drosophila melanogaster* models and studied the molecular consequences. Additionally, we investigated if and how these factors interact with the mitochondrial poly(A) polymerase, MTPAP, as well as with the mitochondrial mRNA stabilising factor, LRPPRC. Our results demonstrate a tight interdependency of mitochondrial mRNA stability, polyadenylation and the removal of antisense RNA. Furthermore, disruption of degradation, as well as polyadenylation, leads to the accumulation of double-stranded RNAs, and their escape out into the cytoplasm is associated with an altered immune-response in flies. Together our results suggest a highly organised and inter-dependable regulation of mitochondrial RNA metabolism with far reaching consequences on cellular physiology.

## Author summary

Although a number of factors have been implemented in the turnover of mitochondrial (mt) DNA-derived transcripts, their exact functions and interplay with one another is not entirely clear. Several of these factors have been proposed to co-ordinately regulate both transcript maturation, as well as degradation, but the order of events during mitochondrial RNA turnover is less well understood. Using a range of different genetically modified D*rosophila melanogaster* models, we studied the involvement of the RNA helicase SUV3, the polynucleotide phosphorylase PNPase, the leucine-rich pentatricopeptide repeat motif-containing protein LRPPRC, and the mitochondrial RNA poly(A) polymerase MTPAP, in stabilisation, polyadenylation, and degradation of mitochondrial transcripts. Our results show a tight collaborative activity of these factors *in vivo* and reveal a clear hierarchical order of events leading to mitochondrial mRNA maturation. Furthermore, we demonstrate that the loss of SUV3, PNPase, or MTPAP leads to the accumulation of mitochondrial-derived antisense RNA in the cytoplasm of cells, which is associated with an altered immune-response in flies.

## Introduction

The turnover of RNA is determined by its rate of synthesis and degradation, and together with the rate of translation, determines the level of gene expression of a given transcript. These processes are not constant and individual RNA species have different half-lives and rates of translation. RNA degradation is frequently used as an important step in regulating gene expression, allowing the cell to rapidly adapt to different physiological demands. This process is fairly well understood in a variety of systems, including the nucleus and cytosol of cells [1], but is less clear in mitochondria.

The mitochondrial genomes (mtDNA) of most metazoans are small, circular, double-stranded, multi-copy genomes, dispersed throughout the mitochondrial network. Gene content and order might vary among species, but the genes are distributed on both strands in most bilaterian animals. Mitochondrial transcription in humans and mice is well defined [2], initiating from two promoters in the regulatory region on either strand of the mitochondrial genome, leading to the generation of long, polycistronic transcripts, which are processed into their individual primary units by enzymes that recognise the gene junctions [2–7].

Just as its mammalian counterpart, the mitochondrial genome of *Drosophila melanogaster* (Dm) encodes for 13 essential subunits of the oxidative phosphorylation (OXPHOS) system, as well as two rRNAs and 22 tRNAs necessary for mitochondrial translation. The mechanism of transcription in Dm is less clear [8]. Dm mtDNA shares none of the sequence elements of the mammalian regulatory region, which in the fly consists almost exclusively of adenine and thymidine residues, giving it the name of the A/T rich region [9]. Its function is not entirely clear, but origins of replication have been associated with this region [10,11], as well as two promoters, similar to the arrangement in mammals [12]. However, the presence of distinct polycistronic transcription units that cover either strand and not originating from the A/T rich region, have led to the suggestion of additional promoter regions in the fly [13,14]. Two members of the MTERF transcription termination family, mTTF and mTERF5, have been suggested to interact with two sequence elements at the boundaries of these transcription units to regulate transcription [13,14], although a role in mtDNA synthesis has also been proposed for these factors [15].

In mammals as well as in the fly, transcription leads to the generation of polycistronic transcripts that need to be processed, and the factors involved are conserved from fly to humans [4,5,7,8,16–19]. The circular nature of mtDNA, with promoters on either strand, means that both sense and antisense transcripts are formed during transcription, but the half-lives of these transcripts are vastly different, despite deriving from the same polycistronic transcript. For instance, antisense RNA species are rarely detected under normal physiological conditions [20–23], and several lines of evidence indicate that processing, maturation and degradation are linked [24–26]. How transcripts are selected for stabilisation or degradation is not known. One factor known to stabilise mitochondrial mRNAs is the leucine-rich pentatricopeptide repeat motif-containing protein, LRPPRC, and its inactivation leads to reduced mitochondrial mRNA steady-state levels in humans [27–29], mice [30,31] and flies [32]. Whether LRPPRC or its Dm ortholog DmLRPPRC1, also known as bicoid stability factor BSF [33], are able to distinguish between coding and non-coding RNAs is unclear, and *in vitro* experiments suggested that human LRPPRC has strong affinity to a broad range of RNA substrates, with lower affinity to poly(A) stretches [29].

Two factors, the ATP-dependent RNA helicase SUV3 and the polynucleotide phosphorylase, PNPase (encoded by PNPT1), have been proposed to form the minimal mitochondrial RNA degrading complex, degrading RNA in a 3′ to 5′ direction [21–23,34]. SUV3 belongs to a highly conserved Ski2 family of DExH-box RNA helicases, with orthologs found in eukaryotes to *Rhodobacter* [35], and PNPase has been shown to both degrade and extend 3′ tails *in vitro* [36]. In plants PNPase regulates polyadenylation-dependent mitochondrial RNA decay [37–39]. Additionally, *in vitro* studies have suggested that SUV3 and PNPase regulate polyadenylation of mitochondrial transcripts by modulating the function of the mitochondrial poly(A) polymerase, MTPAP [40]. This is in agreement with previous work, were we demonstrated that loss of DmSUV3 resulted in reduced polyadenylation but increased steady-state levels of mitochondrial transcripts [41].

Here we studied the relationships of PNPase, SUV3, MTPAP and LRPPRC on mitochondrial RNAs in a series of different Dm models. Our results confirm that PNPase and SUV3 are responsible for mRNA degradation *in vivo* and show that PNPase and SUV3 have opposing effects on polyadenylation. Further, we demonstrate that antisense RNA is not polyadenylated, and we suggest that these RNA species are not recognised by LRPPRC. However, the accumulation of antisense RNA due to the loss of PNPase, SUV3 or MTPAP leads to the accumulation of double stranded mitochondrial RNA, which may leak out into the cytoplasm, affecting other cellular pathways.

## Results

### CG11337 is a mitochondrial protein essential for mitochondrial function in developing flies

**Fig 1:**
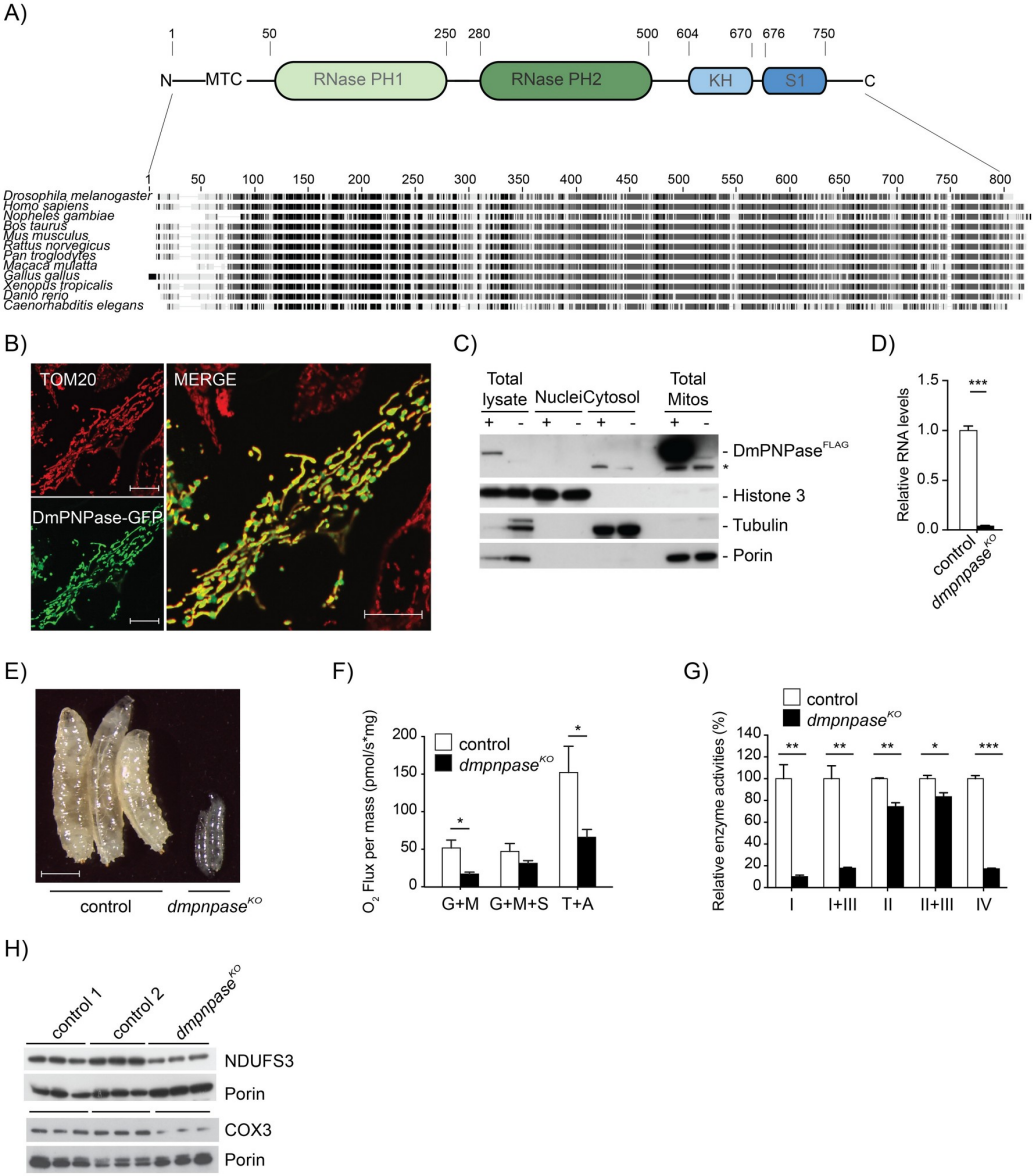
DmPNPase is a mitochondrial protein essential for development in Dm. (**A**) ClustalW alignment of several eukaryotic PNPase sequences, as well as a schematic representation of conserved domains in DmPNPase. (**B**) Confocal analysis of DmPNPase-GFP fusion protein localisation in HeLa cells decorated with TOM20 (red). Scale bars: 5μm (**C**) Western blot analysis of nuclear, cytoplasmic and mitochondrial fractions of DmPNPase-FLAG overexpressing larvae (w;;UAS-*dmpnpase*-flag/daGAL4). Antibodies against the FLAG peptide, tubulin, and histone H3 were used to assess the purity of the fractions; + indicates induced expression of the FLAG tag peptide. An unspecific band is indicated by an asterisk. (**D**) qRT-PCR of *dmpnpase* transcript levels in knockout (w;;*dmpnpase*^KO^/*dmpnpase*^KO^) and controls (w;;) at 4 days after egg laying (AEL). Ribosomal Protein (RP) 49 transcript was used as an endogenous control. (**E**) Body size comparison in controls (w;;) and *dmpnpase*^*KO*^ larvae at 4 days AEL, scale bar size 1mm. (**F**) Mitochondrial oxygen consumption in *dmpnpase*^*KO*^ and wild type larvae (*w*;;). Measurements were performed on an Oroboros oxygraphy, using glutamate, malate and ADP (for complex I driven respiration), then succinate (for complex II driven respiration) and TMPD and ascorbate (for complex IV driven respiration). Error bars indicate the SEM of 9 independent experiments, each measurement was normalised to the protein content of each sample. (**G**) Isolated respiratory chain enzyme activities in *dmpnpase*^*KO*^ and control. Mitochondrial protein extracts from larvae at 4 days AEL were assessed for complex I (NADH coenzyme Q reductase), complex I+III (NADH–cytochrome *c* reductase), complex II (NADH cytochrome *c* reductase), complex III (succinate dehydrogenase), complex II+III (succinate cytochrome *c* reductase) and complex IV (cytochorme *c* oxidase). (**H**) Western blot analysis of mitochondrial encoded COX3 and nuclear encoded NDUFS3 respiratory chain subunits in mitochondrial protein extracts from control and *dmpnpase*^*KO*^ larvae at 4 days AEL. Porin was used as a loading control. (Control 1: w;;, Control 2: w;;*dmpnpase*^KO^/TM6B). All data is represented as mean +/- SEM (***p < 0.001, **p< 0.01, *p< 0.05, n = 5).

A BLAST search against human PNPase or yeast DSS1p ortholog in *Drosophila melanogaster* (Dm) identified *CG11337* as the only candidate, encoding a yet uncharacterised protein, sharing 55.1% identity with human PNPase (Fig 1A). *In silico* analysis predicted a mitochondrial localisation, using TargetP (0.76) or Mitoprot (0.92), and identified the PNPase family-defining RNase PH, KH, and S1 domains [42]. Mitochondrial localisation was confirmed in HeLa cells expressing a GFP-tagged CG11337 fusion protein (Fig 1B), as well as by Western blot analysis of subcellular fractionations of tissue homogenates from Dm larvae expressing a FLAG-tagged CG11337 fusion protein (Fig 1C). Therefore, we suggest CG11337 is the Dm ortholog of PNPase.

In order to explore the involvement of DmPNPase in mitochondrial function, we targeted its locus by CRISPR/Cas9 gene editing technology to generate DmPNPase knockout (*dmpnpase*^*KO*^) flies (see Materials and methods for details). CRISPR/Cas9 editing caused a deletion of 8 nucleotides in exon 2 of the gene, leading to a frame shift and a premature stop codon in the corresponding transcript (S1 Fig). Flies heterozygous for the loss of DmPNPase (w;;*dmpnpase*^KO^/*TM6B*) were viable with no obvious phenotypic changes. Homozygous flies showed a severe reduction in *dmpnpase* transcript levels (Fig 1D) and were larval lethal (Fig 1E). Respirometry measurements of larvae from *dmpnpase*^*KO*^ and control larvae revealed reduced oxygen consumption using both complex I and IV substrates (Fig 1F). This was confirmed by measuring isolated respiratory chain (RC) enzyme activities, demonstrating a combined complex I and IV defect (Fig 1G). *In organello* translation and Western blot analysis using isolated mitochondria from *dmpnpase*^*KO*^ larvae, revealed aberrant translation and reduced steady-state levels of the complex I subunit NDUFS3 and the mitochondrial encoded subunit COX3 upon loss of DmPNPase (S2A Fig and Fig 1H), suggesting instability of the expressed OXPHOS subunits, leading to the observed combined OXPHOS defect observed in the *dmpnpase*^*KO*^ larvae. These findings could be confirmed in flies, where DmPNPase was silenced by RNAi (*dmpnpase*^*KD*^), leading to the same, albeit milder phenotype (S2B–S2G Fig). In conclusion, removal of the DmPNPase gene results in a severe mitochondrial dysfunction and lethality, comparable to the disruption of PNPase in mice [43] and human patients [44–46].

### Modulating the expression of PNPase and SUV3 supports the concept of a mitochondrial degradosome *in vivo*

**Fig 2:**
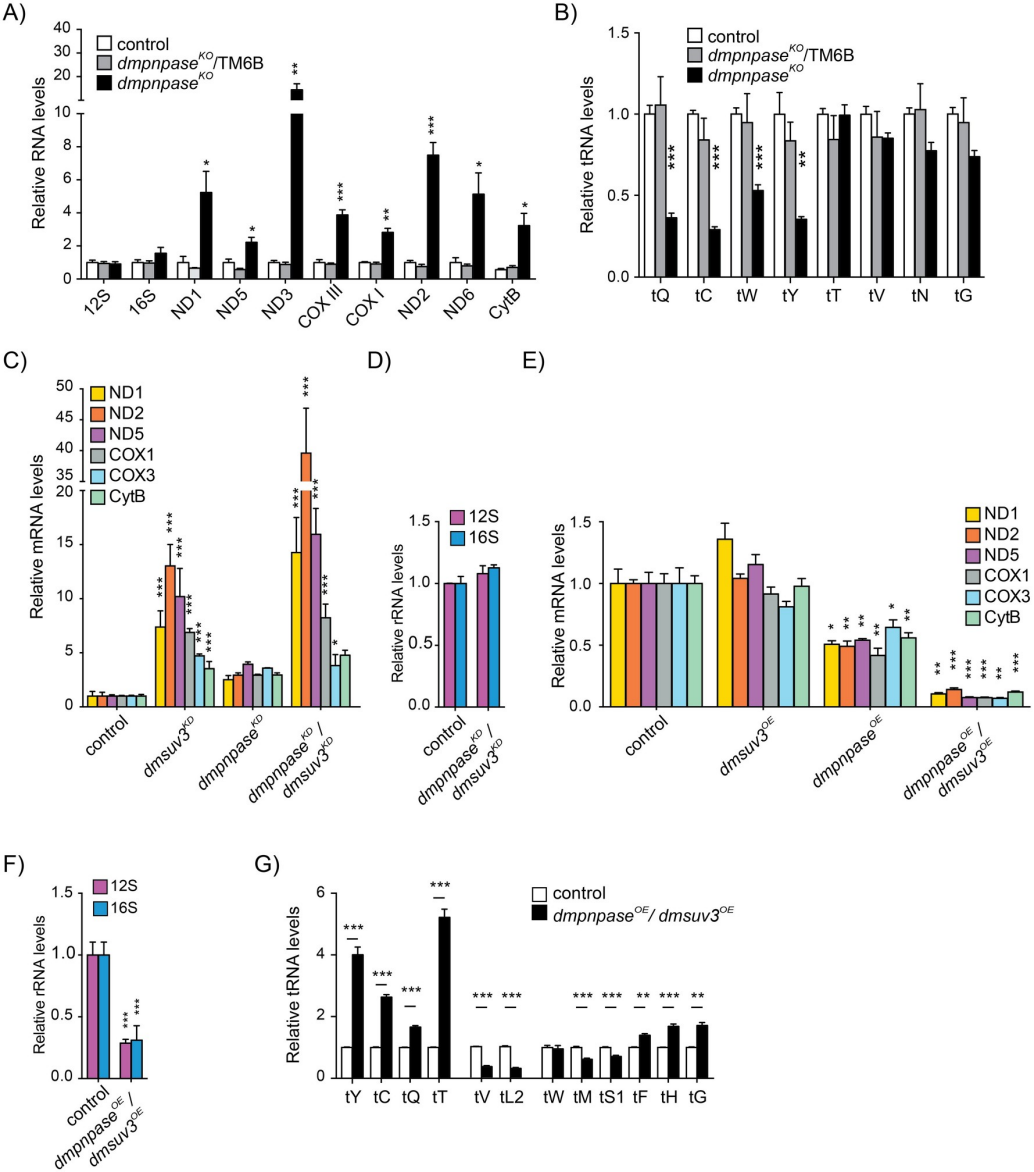
DmPNPase modulates the stability of mt-mRNAs. (**A**) Mitochondrial mRNA steady-state levels in *dmpnpase*^*KO*^ and its controls (control 1: w;;daGAL4/+ and control 2: w;;*dmpnpase*^KO^/TM6B) at 4 days AEL by qRT-PCR. RP49 transcript was used as an endogenous control. (**B**) Quantification of mitochondrial tRNA steady-state levels from Northern blotting analysis in S3B. Cytosolic tRNA^Val^ was used to normalise the quantification. (**C**) Mitochondrial mRNA steady-state levels in DmSUV3^KD^ (w;UAS-*dmsuv3*RNAi/+;daGAL4/+), *dmpnpase*^*KO*^, (w;UAS-*dmpnpase*RNAi;daGAL4/+) *dmpnpase*^*KO*^ /*dmsuv3*^*KD*^ (w;UAS-*dmsuv3*RNAi/UAS-*dmpnpase*RNAi;daGAL4/+) and control (w;;) larvae at 4 days AEL, as determined by qRT-PCR. RP49 transcript was used as an endogenous control. (**D**) Mitochondrial rRNA steady-state levels in *dmpnpase*^*KO*^ /*dmsuv3*^*KD*^ determined by Northern blotting. RP49 transcript was used as an endogenous control for normalisation. (**E**) Mitochondrial mRNA steady-state levels in *dmpnpase*^*OE*^ (w;;UAS-*dmpnpase*/daGAL4), *dmsuv3*^*OE*^ (w;UAS-*dmsuv3*/+;daGAL4/+), *dmpnpase*^*OE*^/*dmsuv3*^*OE*^ (w;UAS-*dmsuv3*/+;UAS-*dmpnpase*/daGAL4) and control (w;;) larvae at 4 days AEL, as determined by qRT-PCR. RP49 transcript was used as an endogenous control. (**F**) Mitochondrial rRNA steady-state levels in *dmpnpase*^*OE*^/*dmsuv3*^*OE*^ larvae determined by qRT-PCR. (**G**) Northern blot quantification of the steady-state levels of mitochondrial tRNAs in *dmpnpase*^*OE*^/*dmsuv3*^*OE*^ and control (w;;) larvae at 4 days AEL. Loading of the gel was normalised and quantified using a probe against cytosolic tRNA^Val^. All data is represented as mean +/- SEM (***p < 0.001, **p< 0.01, *p< 0.05, n = 5).

Loss of DmPNPase *in vivo* led to a general increase in steady-state levels of all analysed mRNAs (Fig 2A and S2G Fig). Northern blot analysis also revealed the presence of smaller species, which we interpret as degradation intermediates (S3A Fig). Steady-state levels of the ribosomal RNA subunits were not affected (Fig 2A), while the levels of some mitochondrial-encoded tRNAs were decreased (Fig 2B and S3B Fig). Increased steady-state levels could be the result of compensatory mechanisms but *in organello* transcription experiments in *dmpnpase*^*KD*^, showed only a mild increase in *de novo* transcription in comparison to mRNA steady-state levels, suggesting that the mRNAs were indeed stabilised (S2F and S2G Fig).

We previously reported that loss of SUV3 in flies leads to pupae lethality with increased mRNA levels and a reduction of mature tRNAs [41]. Together with SUV3, PNPase has been suggested to form the mitochondrial degradosome [21,23] and removing both components simultaneously should therefore lead to additive effects in comparison to removing each individually. Silencing DmPNPase and DmSUV3 by siRNA (see Materials and methods) simultaneously (*dmpnpase*^*KD*^/*dmsuv3*^*KD*^) (S4A and S4B Fig) had a synergistic effect on *mt-nd* transcripts, leading to an up to 30-fold increase in *mt-nd2* steady-state levels (Fig 2C). In contrast, mitochondrial-encoded *cox* and *cytb* transcripts were not further stabilised, in comparison to single DmSUV3 silencing. Silencing of both components of the degradosome had no effect on mt-rRNA steady-state levels (Fig 2D), suggesting differential regulation of the mitochondrial ribosome.

To further confirm the role of both DmSUV3 and DmPNPase in the turnover of mitochondrial transcripts, we generated flies overexpressing DmPNPase (*dmpnpase*^*OE*^), DmSUV3 (*dmsuv3*^*OE*^), or both (*dmpnpase*^*OE*^/*dmsuv3*^*OE*^) (S4A and S4C Fig). Overexpression of DmSUV3 or DmPNPase individually had no or only a mild effect on mRNA steady-state levels, with both fly lines viable (Fig 2E). In contrast, simultaneous overexpression of both components of the degradosome led to larval lethality at the 2nd instar larval stage and a severe reduction of mitochondrial transcript levels, including 12S and 16S rRNA steady-state levels (Fig 2E and 2F). Interestingly, the effects on mt-tRNAs were varied, with steady-state levels increased, unchanged, or decreased (Fig 2G). Taken together, we conclude that *in vivo* DmPNPase and DmSUV3 compose a functional unit that regulates mitochondrial mRNA levels more efficiently than the individual proteins can, supporting the concept of them forming an active complex.

### PNPase and SUV3 have individual and opposite effects on mitochondrial polyadenylation

**Fig 3:**
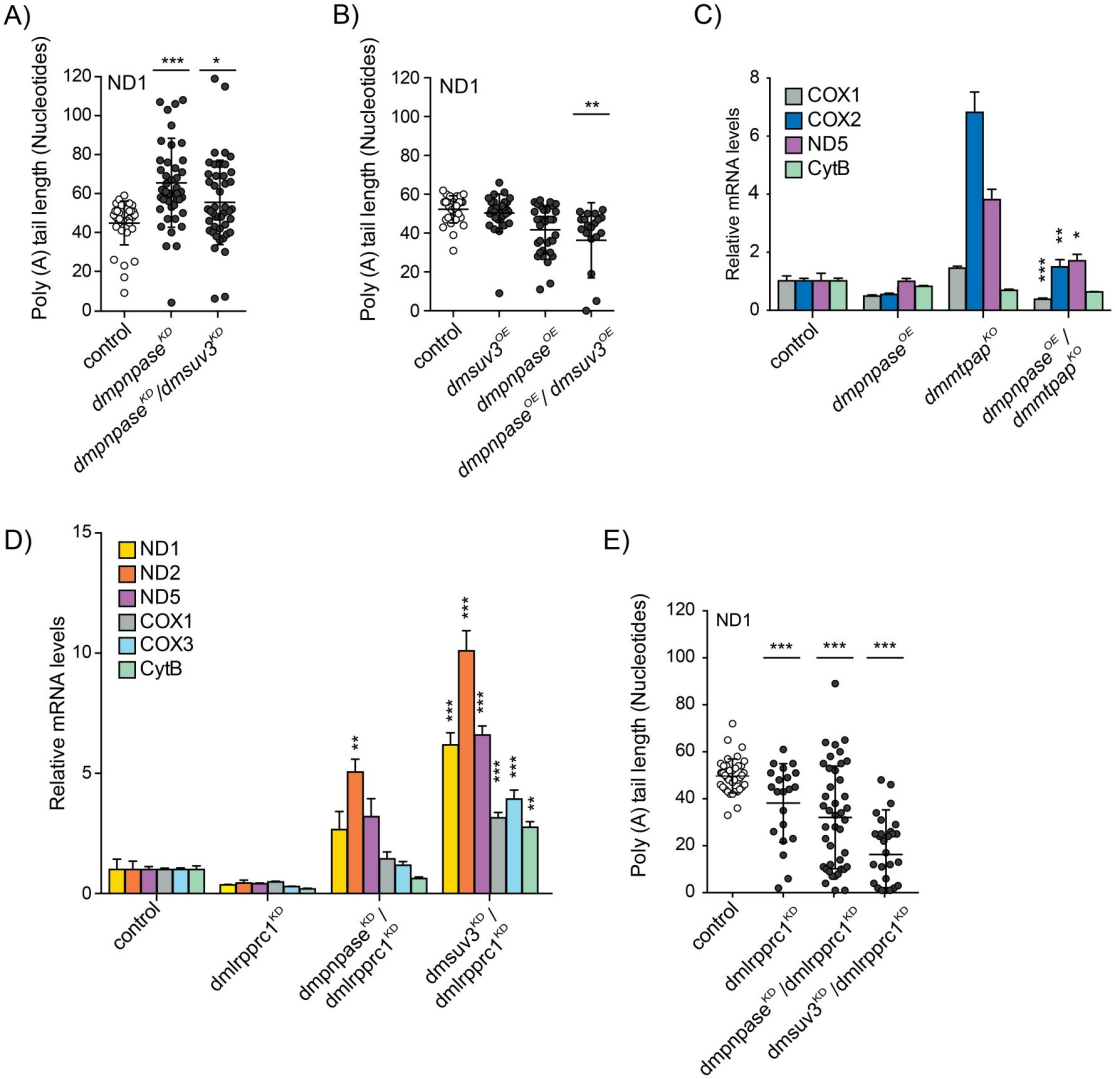
Polyadenylation by mtPAP is not required for degradation and sense strands are protected by LRPPRC. (**A**,**B**) Poly(A) tail length in individually sequenced clones after 3′RACE analysis of ND1 transcripts in *dmpnpase*^*KD*^, *dmpnpase*^*KD*^/*dmsuv3*^*KD*^, *dmsuv3*^*OE*^, *dmpnpase*^*OE*^, *dmpnpase*^*OE*^/*dmsuv3*^*OE*^, and control (w;;) larvae at 4 days AEL. (**C**) Quantification of mitochondrial mRNA steady-state levels of *dmpnpase*^*OE*^ (*dmmtpap*^KO^/FM7;;UAS-*dmpnpase*/daGAL4 or FM7;;UAS-*dmpnpase*/daGAL4 or FM7/Y;;UAS-*dmpnpase*/daGAL4), *dmmtpap*^*KO*^ (*dmmtpap*^KO^/Y;;) *dmpnpase*^*OE*^/*dmmtpap*^*KO*^ (*dmmtpap*^KO^/ Y;;UAS-*dmpnpase*/daGAL4), and control (w;;) larvae at 4 days AEL. Histone was used as loading control. (**D**) Mitochondrial mRNA steady-state levels in *dmlrpprc1*^*KD*^, *dmlrpprc1*^*KD*^/*dmsuv3*^*KD*^, *dmlrpprc1*^*KD*^/*dmpnpase*^*KD*^, and control (w;;) larvae at 4 days AEL, as determined by qRT-PCR. RP49 was used as an endogenous control. (**E**) 3′RACE analysis of poly(A) tails of ND1 transcripts in *dmlrpprc1*^*KD*^ (w;;UAS-*bsf*RNAi#1/daGAL4), *dmlrpprc1*^*KD*^/dmsuv3^KD^ (w;UAS-*dmsuv3*RNAi/+;UAS-*bsf*RNAi#1/daGAL4), *dmlrpprc1*^*KD*^/ *dmpnpase*^*KD*^ (w;UAS-*dmpnpase*RNAi/+;UAS-*bsf*RNAi#1/daGAL4), and control (w;;) larvae at 4 days AEL. All data are represented as mean +/- SEM (***p < 0.001, **p< 0.01, *p< 0.05, n = 5).

PNPase, together with SUV3, has been shown to affect mitochondrial mRNA polyadenylation by either inhibiting or stimulating MTPAP activity *in vitro* [40], but studies *in vivo* have not been performed. In agreement with this, we previously demonstrated that loss of DmSUV3 had a negative effect on poly(A) tail length [41], but whether this was a consequence of mRNA abundance was unclear. We therefore analysed the 3′ ends of mitochondrial ND1 transcripts by 3′RACE, followed by cloning and sequencing in larvae with either depletion (*dmpnpase*^*KD*^/*dmsuv3*^*KD*^) or over-expression (*dmpnpase*^*OE*^/*dmsuv3*^*OE*^) of the mitochondrial degradosome (Fig 3A and 3B). Silencing of DmPNPase alone or together with DmSUV3 resulted in significantly increased poly(A) tail length (Fig 3A), which we confirmed in samples from *dmpnpase*^*KO*^ larvae (S3C Fig). *Dmpnpase*^*KO*^ larvae also presented with an increased amount of shortened tails, which might be attributable to the poor health of the *dmpnpase*^*KO*^ larvae. In contrast, overexpression of the degradosome, which resulted in severe reduction of mRNA steady-state levels, had only a mild effect on poly(A) tail length (Fig 3B). Whether this increased degradation requires an additional deadenylase that first removes the poly(A) tail, is not clear. Nevertheless, our genetic experiments support the notion that DmSUV3 and DmPNPase have opposing effects on the polyadenylation of mitochondrial transcripts also *in vivo*.

### The mitochondrial poly(A) tail is not required for mitochondrial mRNA degradation

Our data thus far demonstrate that both PNPase and SUV3 are necessary and able to degrade mitochondrial mRNAs *in vivo* and that PNPase and SUV3 have opposing effects on polyadenylation. We therefore investigated whether polyadenylation could act as a signal for degradation, similar to the situation in bacteria or plant mitochondria [37]. We previously deleted DmMTPAP by homologous recombination in the fly (*dmmtpap*^*KO*^), demonstrating that polyadenylation was necessary for the integrity of the 3′ terminus of mitochondrial mRNAs [47]. Additionally, mRNA levels were increased in most cases, suggesting that the absence of a poly(A) tail might have prevented their degradation. To test this hypothesis, we crossed flies overexpressing DmPNPase to *dmmtpap*^*KO*^ flies (*dmpnpase*^*OE*^/*dmmtpap*^*KO*^) (S4D Fig) and measured mRNA steady-state levels using Northern blot analysis (Fig 3C and S4E Fig). Mt-mRNA steady-state levels were significantly reduced despite the *dmmtpap*^*KO*^ background, demonstrating that upon overexpression DmPNPase was able to degrade mitochondrial transcripts in the absence of a poly(A) signal. This observation suggests that polyadenylation by MTPAP is not required for the degradation of mitochondrial transcripts, but that other factors might be responsible for regulating mRNA stabilisation.

### LRPPRC protects sense strands of mRNA from the mitochondrial degradosome

One such factor is LRPPRC, known to stabilise mitochondrial mRNAs [48,49]. Additionally, we and others previously demonstrated that LRPPRC is required for sufficient polyadenylation [31,32,50]. In order to investigate the relationship between PNPase, SUV3 and LRPPRC *in vivo*, and to probe whether the degradosome is responsible for the degradation of mRNAs in the absence of LRPPRC, we generated flies with depleted DmPNPase or DmSUV3 in addition to DmLRPPRC1 [32] (*dmlrpprc1*^*KD*^/*dmpnpase*^*KD*^ or *dmlrpprc1*^*KD*^/*dmsuv3*^*KD*^) (S4F Fig). Decreasing either PNPase or SUV3 in combination with LRPPRC stabilised mRNA steady-state levels in comparison to *dmlrpprc1*^*KD*^ alone (Fig 3D), suggesting that indeed, LRPPRC functions as a physical barrier, protecting mRNAs from degradation by the degradosome. Surprisingly though, silencing of DmPNPase did not restore poly(A) tail length in the absence of DmLRPPRC1 (Fig 3E). Thus, the degradosome is not responsible for the reduced polyadenylation, but rather MTPAP has inefficient processivity in the absence of LRPPRC. This is consistent with previous observations, where LRPPRC was able to stimulate MTPAP processivity *in vitro* [50].

### Antisense RNAs are oligoadenylated but not polyadenylated

**Fig 4:**
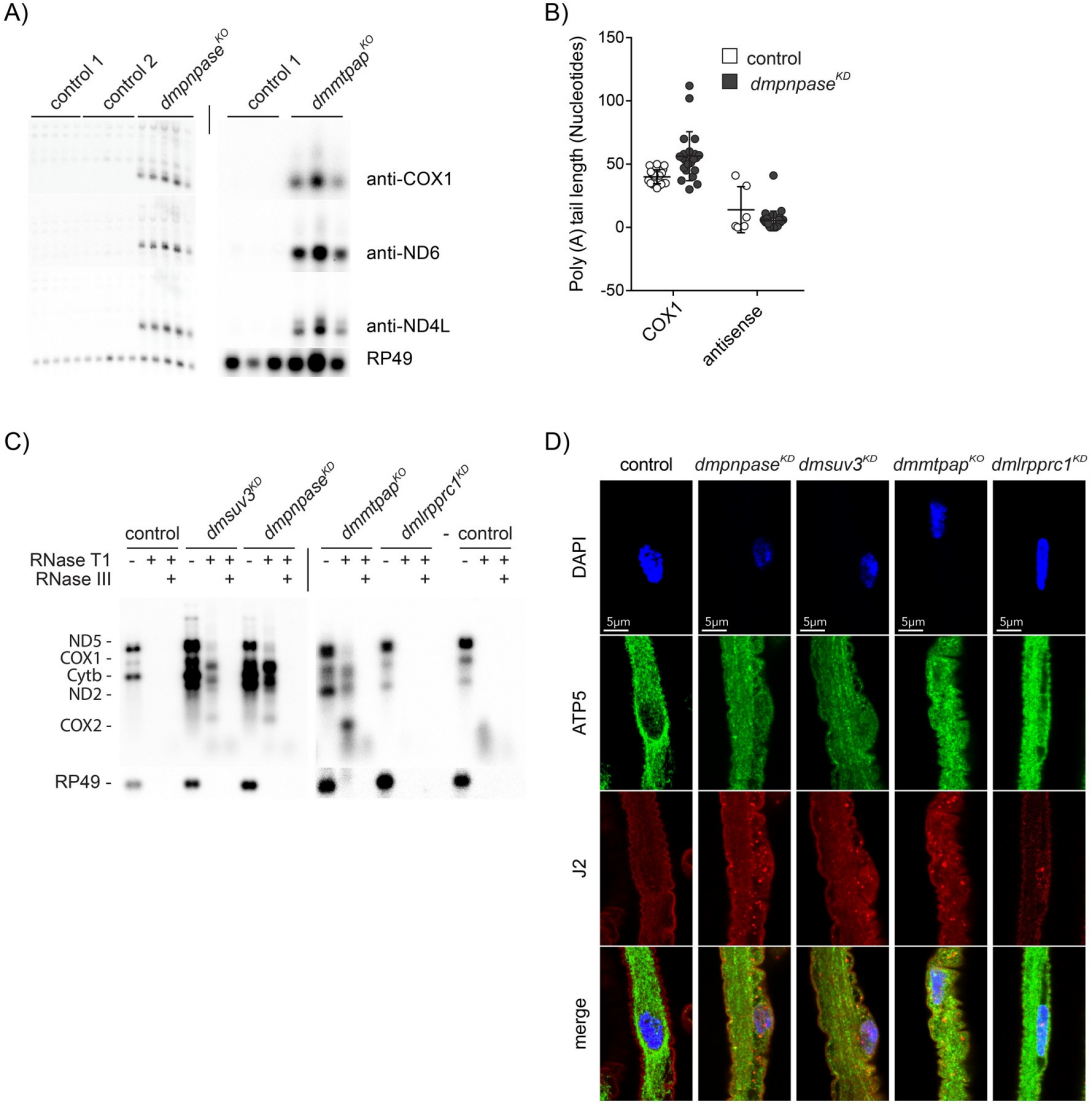
Mitochondrial dRNAs accumulate and localise to the cytoplasm in the absence of mtPNPase, SUV3 and mtPAP. (**A**) Northern blot analysis by formaldehyde-agarose gel electrophoresis with total RNA isolated from 4 day AEL larvae. The signals were detected using double stranded DNA probes and antisense strand oligonucleotide probes. Nuclear encoded RP49 was used as a loading control. (**B**) Poly(A) tail length of COX1 transcripts and COX1 antisense strands in individually sequenced clones after 3′RACE experiments in *dmpnpase*^*KD*^ (n = 22 and n = 31, respectively), and wild type control (w;;, n = 23 and n = 5, respectively) larvae at 4 days AEL. (**C**) Northern blots of total RNA isolated from larvae at 4 days AEL of controls, *dmsuv3*^*KD*^, *dmpnpase*^*KO*^, *dmmtpap*^*KO*^, and *dmlrpprc1*^*KD*^ treated with different RNases as indicated. The blots were probed with 5 different probes against mitochondrial transcripts and with RP49 as a loading control. (**D**) Immunostaining of dsRNA with J2 antibodies (red) in dissected brains from larvae of control, *dmpnpase*^*KO*^, *dmsuv3*^*KD*^, *dmmtpap*^*KO*^, and *dmlrpprc1*^*KD*^. Mitochondria are stained with ATP5a antibodies (green) and nuclei are stained with DAPI (blue).

Processing of the polycistronic transcripts leads to the formation of non-coding anti-sense RNA, which is rapidly removed under normal conditions [20–23]. However, the mechanism for this selective removal is unknown. PNPase and SUV3 have been suggested to degrade antisense RNA in cells and we and others previously reported that silencing of DmSUV3 resulted in the accumulation of antisense RNA [41]. In agreement, loss of DmPNPase also resulted in stabilisation of antisense RNA (Fig 4A). More surprisingly though, loss of polyadenylation due to the absence of DmMTPAP also led to the accumulation of antisense RNA (Fig 4A). Previous work in mice, using immunoprecipitation (PAR-CLIP) against LRPPRC, followed by high-throughput sequencing analysis, suggested that LRPPRC does not bind to antisense RNA [51]. If LRPPRC does not bind antisense RNA, polyadenylation of antisense should be reduced due to the lack of LRPPRC. We therefore analysed the 3′ ends of transcripts antisense to *cox1* in control and *dmpnpase*^*KO*^ larvae and found that these antisense transcripts had a mean length of only 5 adenines, suggesting that antisense RNA is not extensively polyadenylated, possibly due to the lack of LRPPRC stimulation (Fig 4B). This suggests that antisense RNA is quickly removed by DmPNPase and SUV3 prior to protection by LRPPRC, although MTPAP is still able to initiate oligoadenylation, i.e. the addition of only a few adenosines. Sense mRNAs, on the other hand, are protected by LRPPRC, polyadenylated and maintained intact in order to be translated and the resulting proteins being assembled.

### Mitochondrial dsRNAs are formed in the absence of PNPase, SUV3 and MTPAP

The accumulation of antisense RNA leads to the possibility of the formation of intermolecular double strand RNA (dsRNA), where sense mRNAs hybridise to their antisense counterpart. Indeed, mitochondrial dsRNAs have already been observed previously *in vitro* [20,52], and their existence in cells was recently demonstrated and even suggested to be able to be released into the cytosol under conditions of perturbed mtRNA degradation [53]. To investigate whether dsRNA also accumulates in flies, we isolated RNA from larvae lacking DmSUV3, DmPNPase, or DmMTPAP and treated the samples with RNases specific for single- (RNase T1) or double-stranded (RNase III) RNA, followed by Northern blot analysis to several mitochondrial targets (see Materials and methods). The accumulation of antisense RNA did indeed lead to the formation of RNA species in the absence of DmPNPase or DmSUV3, which were resistant to RNaseT1-treatment, but which could be removed by RNase III, a nuclease with preferentially dsRNA as substrate (Fig 4C). Surprisingly, *dmmtpap*^*KO*^ larvae also accumulated dsRNA, suggesting that dsRNA can be formed by a range of disrupted processes in mitochondrial RNA metabolism. As expected, larvae lacking DmLRPPRC1 (*dmlrpprc1*^*KD*^) did not show any signs of dsRNA (Fig 4C).

### Mitochondrial dsRNA escapes into the cytoplasm in absence of PNPase, SUV3 and MTPAP

**Fig 5:**
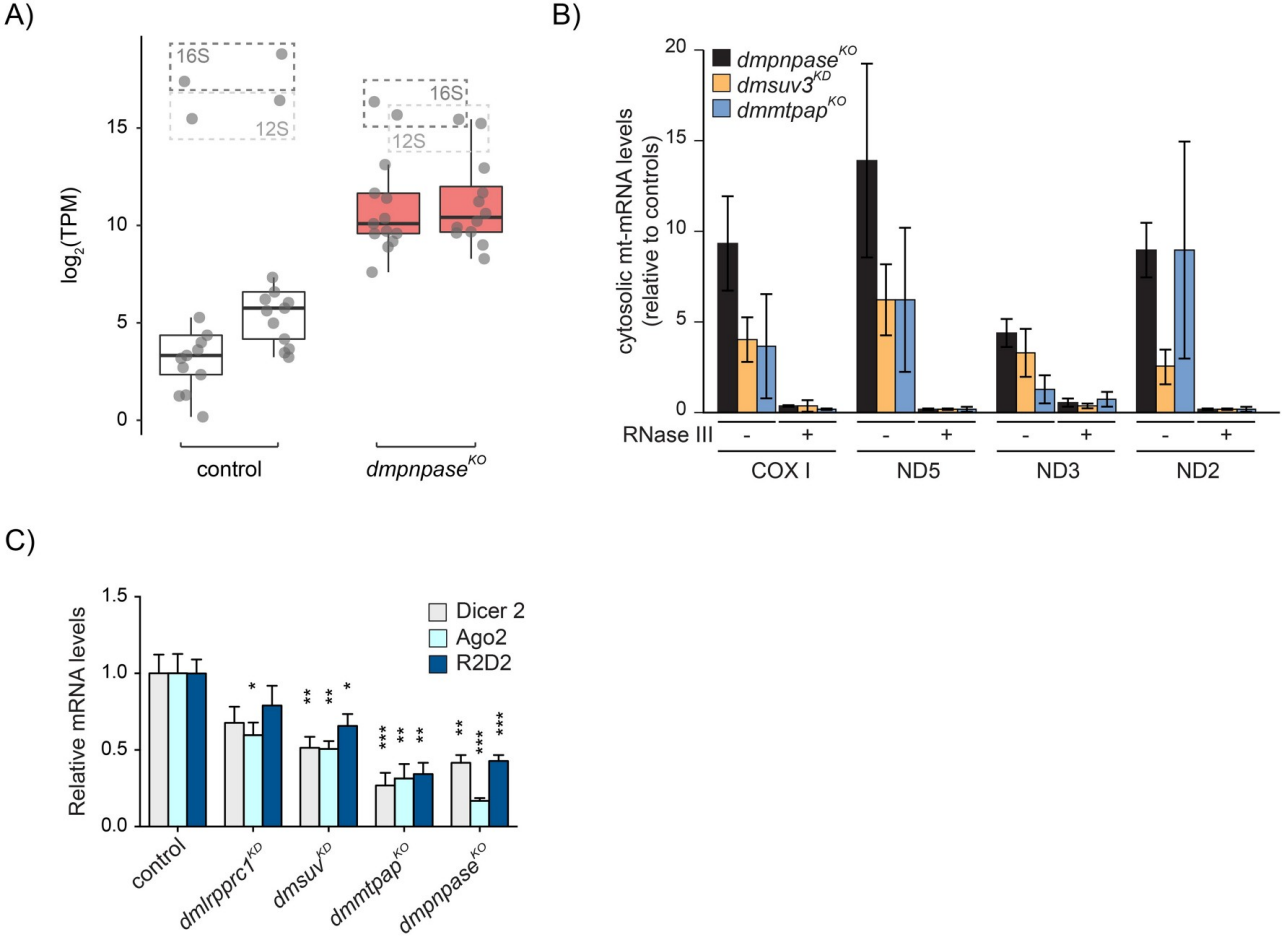
Cytoplasm-localised mitochondrial dsRNAs affect the cellular antiviral response pathways. (**A**) Boxplot showing normalised transcripts per million (TPM) values of double-stranded mitochondrial transcripts within the cytosolic fraction of control and *dmpnpase*^*KO*^ fly models (n = 2). (**B**) qRT-PCR analysis of mtCOX1, mtND5, mtND3, and mtND2 in cytosol, fractionated from larvae of controls, *dmpnpase*^*KO*^, *dmsuv3*^*KD*^, and *dmmtpap*^*KO*^ and treated with RNase III. Data are represented as mean +/- SEM. (**C**) qRT-PCR analysis of Dicer2, Ago2, and R2D2 transcript levels in larvae of controls, *dmlrpprc1*^*KD*^, *dmpnpase*^*KO*^, *dmsuv3*^*KD*^, and *dmmtpap*^*KO*^. All data are represented as mean +/- SEM (***p < 0.001, **p< 0.01, *p< 0.05, n = 5 for *dmlrpprc1*^*KD*^, *dmpnpase*^*KO*^, and *dmsuv3*^*KD*^. n = 3 for *dmmtpap*^*KO*^).

Recently, Dhir and colleagues demonstrated that the disruption of PNPase or SUV3 in cells can lead to the formation of dsRNA, and that this dsRNA can be released from mitochondria in the absence of PNPase [53]. We therefore performed immunohistochemistry on isolated brains from *dmpnpase*^*KO*^, *dmsuv3*^*KD*^, *dmmtpap*^*KO*^, and *dmlrpprc1*^*KD*^ larvae, using the antibody J2 that recognises dsRNA. Our results showed a punctuate cytosolic pattern in *dmpnpase*^*KO*^, *dmsuv3*^*KD*^ and *dmmtpap*^*KO*^ larval brains, while control or *dmlrpprc1*^*KD*^ larvae showed a reduced signal (Fig 4D). In none of the models though, the J2 signal clearly co-localised with mitochondria, as determined by counterstaining against the mitochondrial ATPase subunit, ATP5a, which is likely due to the J2 antibody unable to penetrate mitochondria in the experimental conditions. Nevertheless, these results indicate that dsRNAs can accumulate in the cytosol of fly models lacking *dmpnpase*^*KO*^, *dmsuv3*^*KD*^, or *dmmtpap*^*KO*^. We next performed J2-immunoprecipitation-based dsRNA sequencing (dsRNA-seq) on sub-cellular fractions from *dmpnpase*^*KO*^ larvae in order to identify the nature of these dsRNAs (Fig 5A). Purity of our preparations were confirmed by Western blot analysis (S5A Fig). When normalised to control samples, dsRNA was extensively enriched in both mitochondrial and cytosolic fractions in *dmpnpase*^*KO*^ samples (Fig 5A, and S5B and S5C Fig), indicating that mitochondrial-derived dsRNA can be released from mitochondria in flies. To confirm the exclusivity of mitochondrial dsRNA leakage in the absence of PNPase, we investigated the accumulation of dsRNA in *dmpnpase*^*KO*^, *dmsuv3*^*KD*^, and *dmmtpap*^*KO*^ samples with qRT-PCR for mitochondrial transcripts in cytosolic fractions. To our surprise, we observed a significant increase of mitochondrial-derived RNA, sensitive to the dsRNA-specific endoribonuclease RNase III, in cytoplasmic fractions from all 3 models (Fig 5B), suggesting that the dsRNAs observed by immunostaining are indeed of mitochondrial origin. The accumulation of mitochondrial-derived dsRNA in the cytosol could be a consequence of altered mitochondrial morphology. We therefore investigated mitochondrial morphology in the ventral nerve cord of larvae deficient in PNPase, SUV3, MTPAP, or LRPPRC as well as in larvae overexpressing both PNPase and SUV3 by confocal microscopy [54]. However, we observed no gross difference in any of the mutant fly lines to control larvae (S6 Fig).

Mitochondrial-derived dsRNAs in the cytoplasm have recently been shown to activate the MDA5-driven antiviral signalling pathway in human cell lines [53]. Although flies and humans do not share the same antiviral responses, we analysed mRNA levels of the corresponding antiviral response genes Dicer2, Ago2, and R2D2 in flies [55,56]. Transcript levels of all three factors were significantly decreased in *dmpnpase*^*KO*^ and *dmmtpap*^*KO*^ larvae, with a milder response in *dmsuv3*^*KD*^. Silencing of *dmlrpprc1*, which has an OXPHOS defect but did not leak dsRNA into the cytoplasm, resulted in a trend for reduced transcript levels, with only Ago2 levels being significantly reduced (Fig 5C). Dicer2, R2D2, and Ago2 have been shown to be essential for the fly antiviral defense [57–59], suggesting that the loss of factors involved in mitochondrial RNA metabolism can result in a hypersensitivity to viral infections. Together, our results suggest that dsRNA can escape the mitochondrial matrix upon disruption of several factors involved in the turnover of mitochondrial RNA.

## Discussion

The mechanisms involved in mitochondrial RNA turnover remain poorly understood, and a number of factors have been associated with the degradation of mitochondrial RNAs. Two of these factors, PNPase and SUV3, have been extensively studied, and have been suggested to constitute the mitochondrial degradosome. The majority of these studies have been performed *in vitro* or in cell culture and their *in vivo* role is therefore not always clear. Additionally, many of these studies only investigated a single protein in isolation, but it is increasingly becoming clear that many of these factors work cooperatively. We therefore used a combination of transgenic Dm models to genetically address the interactions of the mitochondrial degradosome, and how PNPase and SUV3 affect the functions of the mitochondrial mRNA stabilising protein, LRPPRC, or the mitochondrial poly(A) polymerase, MTPAP.

We provide *in vivo* evidence that CG11337 is the fly orthologue of PNPase, and its disruption has wide ranging consequences on mitochondrial RNA metabolism and cell function. PNPase has phosphorolytic 3′ to 5′ exoribonuclease activity and its overexpression in Dm led to reduced mitochondrial mRNA steady-state levels, while deletion of DmPNPase led to the accumulation of mitochondrial mRNAs, suggesting a direct role in mRNA turnover. These effects were amplified when both factors of the proposed mitochondrial degradosome were either increased or decreased, further supporting their complementary function. We were surprised, though, that silencing of DmSUV3 had a stronger effect on steady-state levels than DmPNPase knockdown, suggesting that residual PNPase protein levels are highly active, but are dependent on SUV3. In this case, a strict regulation of PNPase function is required to prevent unwanted degradation. However, the mechanism of such regulation remains unknown. Interestingly, effects on rRNAs and tRNAs were less pronounced, suggesting that PNPase and SUV3 are not involved in their turnover and changes might be a secondary response.

**Fig 6:**
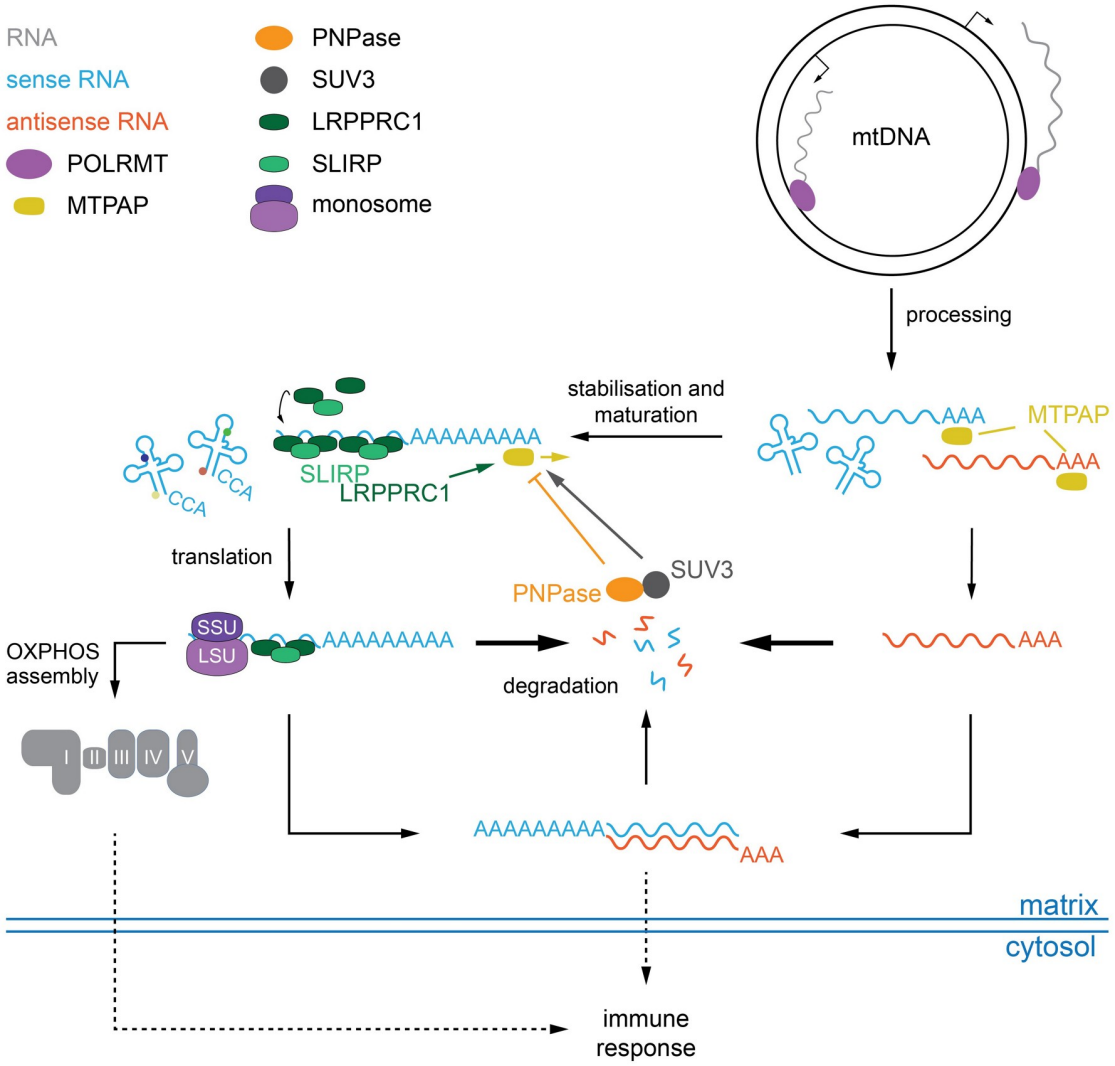
Schematic diagram of the proposed degradation pathway of mitochondrial transcripts. Transcription by POLRMT of polycistronic transcripts is initiated from two promoters on either strand of the mitochondrial genome (mtDNA). Processing into individual, immature transcripts is performed by the RNase P and RNase Z complexes (not indicated), before MTPAP adds short poly(A) extensions to the 3′ ends of both sense and antisense RNAs and potentially also to selected tRNAs. Coding RNAs are stabilised by the LRPPRC/SLIRP complex, matured and fully polyadenylated in an MTPAP/LRPPRC/SUV3-dependent manner. An unknown mechanism targets antisense RNAs directly for degradation by the PNPase/SUV3 complex. In the absence of degradation, or polyadenylation, antisense RNAs are stabilised allowing for the formation of dsRNAs, which are expelled from the mitochondrial matrix by an unknown mechanism. The accumulation of mitochondrial-derived nucleic acids and/or an OXPHOS dysfunction triggers an antiviral immune response.

The current model of mitochondrial RNA turnover (Fig 6) suggests that the leucine-rich PPR motif-containing protein, LRPPRC stabilises mitochondrial mRNAs, and its disruption leads to a rapid depletion of mitochondrial mRNAs in flies [32], mice [31], and cells [60]. Simultaneous loss of DmLRPPRC1 and the degradosome restored mRNA steady-state levels, supporting the role of SUV3 and PNPase as the main responsible factors for the degradation of transcripts not protected by LRPPRC. Besides degrading mRNAs, loss of the degradosome also resulted in the accumulation of antisense RNA, suggesting that PNPase and SUV3 are also responsible for degrading these RNAs. This is in agreement with recent results that observed the accumulation of antisense RNA upon depletion of the degradosome [34,53]. Interestingly, MTPAP knockout larvae also accumulated antisense RNA, suggesting that polyadenylation might be required for removal. We did indeed observe short poly(A) tails on antisense transcripts, but whether these are required for degradation remains to be investigated. On the other hand, overexpression of DmPNPase could significantly reduce mRNA levels in the absence of polyadenylation, suggesting that transcripts lacking a poly(A) tail are more sensitive to increased PNPase levels, or that polyadenylation might not be a prerequisite for degradation.

Several lines of evidence suggest that LRPPRC is required for full polyadenylation of mature mitochondrial transcripts, by stimulating MTPAP [31,32,50,51,60]. Additionally, PNPase, SUV3 and MTPAP have been proposed to form a transient complex to modulate poly(A) tail length in response to cellular energy demands [40]. However, the way how these factors work together *in vivo* has not been investigated. Our data demonstrate that LRPPRC is required for full polyadenylation *in vivo* and that PNPase and SUV3 have opposing effects on MTPAP processivity. Loss of both LRPPRC and PNPase did not restore poly(A) tail length, supporting the idea that shortening of poly(A) tails is not a consequence of degradosome function but rather due to absence of stimulation of MTPAP by LRPPRC, as suggested by *in vitro* studies [50].

The occurrence of short poly(A) tails on antisense RNA suggests two things. First, antisense RNAs are not recognised by LRPPRC, because even when stabilised due to the disruption of PNPase, they are not fully polyadenylated. Further, it also suggests that MTPAP can initiate adenylation in the absence of LRPPRC, but full polyadenylation requires LRPPRC interaction. The notion that LRPPRC does not bind antisense RNA is supported by recent PAR-CLIP experiments against LRPPRC that did not identify unprocessed or antisense RNA [51]. What distinguishes sense from antisense RNA is not clear, but recently the quasi-RNA recognition motif (qRRM) protein GRSF1 has been shown to recognise and—together with PNPase and SUV3—resolve and degrade G-quadruplex structures, which predominantly occur in non-coding mitochondrial RNAs [34]. GRSF1 is not conserved outside of vertebrates and thus additional mechanisms are likely to distinguish different RNA species, although structural elements in the RNAs are likely. In addition, the presence of a start codon on sense transcripts and consequently the recruitment of regulatory proteins and translational activators might rapidly target the transcripts for translation and hence make them escape the degradation apparatus. Further, it is possible that the formation of dsRNA prevents LRPPRC binding, and thus, full polyadenylation. Nevertheless, we confirm the involvement of both PNPase and SUV3 in the degradation of both mRNAs and antisense RNAs *in vivo*, and that both factors are able to modulate polyadenylation of mitochondrial mRNAs. Further, we reveal that this influence acts only on mRNA associated with LRPPRC, but that the stimulatory effect of LRPPRC on polyadenylation occurs after initiation of adenylation by MTPAP. Thus, we provide a hierarchical order of mitochondrial transcript maturation, where immature coding transcripts are first adenylated by MTPAP, followed by stabilisation by LRPPRC and full polyadenylation in a SUV3-dependent manner. Additionally, our data demonstrate that non-coding antisense transcripts, which are routinely generated during mitochondrial transcription, are recognised by MTPAP, but not by LRPPRC and are therefore rapidly degraded in a SUV3/PNPase-dependent manner (Fig 6).

Our results indicate that the accumulation of mitochondrial antisense RNA can lead to the formation of dsRNA species, by disrupting PNPase, SUV3, as well as MTPAP. DsRNA in mitochondria was first described by Young and Attardi [52], but recently shown to be released into the cytosol in the absence of PNPase in mammalian cells, where this release could increase interferon B1 (IFNB1) levels via the RNA sensor MDA5 and subsequently via MAVS [53]. Various reports suggest that the accumulation of mitochondrial-derived nucleic acids in the cytosol can trigger the innate immune response in mammals [53,61–64]. In human cells, the release of dsRNA seems to be restricted to the loss of PNPase, as silencing of human SUV3 leads to the accumulation of dsRNA but not their release into the cytosol [53]. In contrast, we show that the release of mitochondrial dsRNA can be caused by a variety of cellular stresses *in vivo*, including the loss of PNPase, SUV3 or MTPAP, suggesting that the release of dsRNA might be a downstream consequence to a mitochondrial defect.

Neither the MDA5-MAVS signalling pathway, nor IFNB1 are conserved in Dm. Instead, Dm use Dicer2, which together with Ago2 and R2D2, constitutes the main RNA sensor in antiviral signalling [57–59]. We demonstrate a down regulation of these antiviral response factors, suggesting an increased sensitivity to viral infections. Dicer2 processes dsRNA into 21nt duplex siRNA, and the Dm models described here accumulated long stretches of mitochondrial-derived dsRNA into the cytoplasm, which is consistent with the reduced levels of Dicer2 observed in these lines. It is likely that the mitochondrial dysfunction, observed in these flies, has at least a partial impact on the ability to respond to viral infections, although it is not clear whether the release of mitochondrial-derived dsRNA is the primary signal. How and why dsRNAs are released into the cytoplasm is not known, but we failed to observe any obvious morphological changes in the mitochondrial network in the brains of the investigated Dm models. Nevertheless, there is growing evidence for an important role for mitochondrial function in human immunity [61,65]. The altered immune responses to mitochondrial defects observed here could explain why some patients with mitochondrial disease respond catastrophically to infections [64,66]. However, it will also be important to understand if and in what way the existence of dsRNA inside the mitochondrial network can affect mitochondrial function.

## Materials and methods

### *Drosophila* stocks, maintenance and hatching rates

All genomically engineered fly lines were maintained and experiments performed at 25°C and 60% humidity on a 12h:12h light:dark cycle and fed on a standard yeast–sugar–agar (10-5-1) medium. All fly stocks were backcrossed for at least 6 generations into the white Dahomey Wolbachia-free background (*w*). For *in vivo* knockdown of *dmpnpase*, a w;UAS-*dmpnpase*-RNAi; line was obtained from the Vienna Drosophila Resource Centre (VDRC, #108198). For *in vivo* knockdown of *dmsuv3* a w;UAS-dmsuv3-RNAi; line was obtained from the National Institute of Genetics (NIG-Fly, Japan, #9791R-2).

Ubiquitous knockdown of *dmsuv3*, *dmpnpase* or double mutants was achieved by crossing the UAS-RNAi lines to the driver line daughterless GAL4 (w;;daGAL4). The balancer fly line stocks (+;CyO/Gla;+); and (+;+;TM3,Sb Ser/TM6B) were used to generate all the double mutants line stocks. Constructs for the generation of fly lines overexpressing DmPNPase or DmSUV3 were sent for embryo injection to BestGene (California, USA).

For adult hatching rate measurements, flies were allowed to lay eggs on grape juice agar plates for 8h. Then, the eggs were collected and transferred to vials (100 eggs/vial) with yeast–sugar–agar medium. Hatching was recorded daily. At least five biological replicates were performed per genotype.

### Generation of DmPNPase KO using CRISPR/Cas9

The generation of the PNPase^KO^ line (*dmpnpase*^*KO*^) was performed as previously described [67], using the transgenic fly line *nos*-*cas9* (Bloomington stock centre: 54591). Genomic DNA from the *cas9*-fly strain was used to amplify and sequence the *dmpnpase* gene for the selection of CRISPR targets. The *pnpase* sequence was submitted to CRISPR Optimal Target Finder [68] and two target sites (5′: GACCTTCAGTTCCGGCCGCC and 3′: ATCTAATATTCTGGACATC) with the lowest off-target cleavage score were selected. The cloning strategy of the gRNAs into pCFD4 plasmid (AddGene plasmid 49411) was followed as in [67]. Briefly, the *dmpnpase* target sites were included in forward and reverse primers with homology to pCFD4, and used on a PCR using the plasmid as template. PCR products were then cloned into the BbsI-digested pCFD4 vector by Gibson assembly. The pCFD4 plasmid containing the gRNAs was injected into embryos in Bestgene to generate a gRNA expressing fly line. To induce germ line cleavage, the transgenic *nos-cas9* virgin females were crossed to gRNA–expressing males. Resulting chimaeras were individually crossed to TM3/TM6B balancer flies, and the offspring was screened by for deletions at the *dmpnpase* locus. Individual candidate flies were again crossed to the TM3/TM6B balancer line, followed by screening for homozygous lethality in the offspring of intercrosses. PCR screening was performed as follows. The wings of candidate flies were incubated at 37°C for 45 min in 50 μl of freshly prepared adult fly homogenisation buffer (10 mM Tris-HCl,pH 8.2, 25 mM NaCl, 1 mM EDTA, 0,2 μg/μl proteinase K) and 2 μl of the homogenate was used for the PCR, subsequent screening and sequencing. The obtained *dmpnpase*^*KO*^ lines were backcrossed for 6 generations to a w^Dah^ background to remove possible off-target effects.

### Constructs

Full-length *dmpnpase* cDNA was obtained from the Drosophila Genomics Resource Centre (LD03255). *dmpnpase* cDNA was cloned into pEGFP-N3 plasmid (Clontech) to generate a DmPNPase-GFP fusion construct. To generate a *dmpnpase-Flag* fusion construct the cDNA was amplified with a reverse primer carrying an in-frame FLAG tag. The cDNAs were cloned into pUAST plasmid to generate the *dmpnpase*-overexpressing fly lines. Primers used for the cloning of *dmpnpase* are listed in S1 Table.

### Cell culture, transfection and Dm cellular sub-fractionations

HeLa cells were cultured in high-glucose DMEM (Thermo Scientific) supplemented with 10% foetal bovine serum (BSA) at 37°C in a 5% CO_2_ atmosphere. For co-localisation studies, HeLa cells were transfected with a *dmpnpase*-GFP fusion construct, using a calcium phosphate transfection kit (Sigma-Aldrich), following the manufacturer’s instructions. 48 hours after transfection HeLa cells were fixed with 4% PFA and decorated with anti-TOM20 antibody (Santa Cruz, sc-11415). Images were obtained with a Nikon Confocal Microscope at the Live Cell Imaging Unit, Karolinska Institutet.

Dm nuclear, cytoplasmic and mitochondrial fractions were prepared from larvae four days after egg lay (AEL) by differential centrifugation as previously described [69]. Purity was assessed by Western blotting, using primary antibodies against Histone H3 (Santa Cruz Biotechnology, dilution 1:200), Complex I-subunit NDUFS3 (Mitoscience MS112, dilution 1:1000), tubulin (Sigma, dilution 1:1000), porin (Abcam, dilution 1:5000) and FLAG (Sigma, dilution 1:1000). Protein bands were visualised with Clarity Western ECL substrate (Bio-Rad).

To obtain Percoll purified mitochondria, 3 mg of crude mitochondria were layered on 20% Percoll in STE buffer (250 mM sucrose, 5 mM Tris, 2 mM EGTA, pH 7.4) and centrifuged at 40,000g for 30 min at 4°C in a Beckman SW41 rotor. The pure mitochondrial fraction was pipetted off the bottom of the tube and washed twice in STE buffer at 7,000g to dilute residual Percoll. Mitochondria were pelleted at 9,000g, flash-frozen in liquid nitrogen and stored at -80°C until further analysis.

### Biochemical evaluation of respiratory chain function

For mitochondrial isolation third-instar larvae were homogenised in ice-cold isolation buffer STE (250 mM sucrose, 5 mM Tris, 2 mM EGTA, pH 7.4) + 5% BSA (w/v), using a Dounce homogeniser. Cellular debris was pelleted at 1,000g for 5 min and supernatants were transferred to new tubes. Mitochondria were washed twice at 3,000g and the final mitochondrial fraction was pelleted at 7,000g and resuspended in STE buffer. For determination of the activities of respiratory chain complexes, protein concentration of the mitochondrial preparations was determined using a Qubit fluorometer and mitochondria were resuspended in 250 mM sucrose, 15 mM KH_2_PO_4_, 2 mM MgAc_2_, 0.5 mM EDTA and 0.5 g/L BSA, pH 7.2. Biochemical activities of the respiratory chain complexes were determined as previously described [70].

For oxygen consumption measurements, larvae at 4 days AEL were dissected and resuspended in Mir05 respiratory buffer (110 mM sucrose, 10 mM KH_2_PO_4_, 3 mM MgCl_2_, 0.5 mM EGTA, 60 mM lactobionic acid, 20 mM taurine, 20 mM HEPES, BSA 1 g/l pH 7.1). Oxygen consumption was measured at 25°C using an oxygraph chamber (OROBOROS). Larvae were permeabilised with 0.02 mg/ml digitonin. Complex I-dependent respiration was assessed by adding the substrates malate (2 mM) and glutamate (10 mM), followed by addition of 2.5 mM ADP. Complex I-independent respiration was measured in the presence of 0.5 μM rotenone. Complex II-dependent respiration was measured using 10 mM succinate (SUCC). Complex III was inhibited with 2.5 μM antimycin A. Finally, Complex IV activity was measured by addition of 2 mM ascorbate and 0.5 mM N,N,N’,N’-tetramethyl-p-phenylenediamine dihydrochloride (TMPD) and subsequent addition of 1mM potassium cyanide. Assessment of the quality of the measurements was performed by adding 10 μM cytochrome *c* to the samples. Finally, the protein content was determined by the Bradford method (BioRad) in order to normalise the oxygen consumption flux to protein content.

### Blue-Native polyacrylamide gel electrophoresis (BN-PAGE) and in-gel activity assays

BN-PAGE and in-gel staining for complex I and IV activities was performed on isolated mitochondria. Mitochondria were pelleted and lysed in digitonin buffer (1% digitonin, 20 mM Tris pH 7.4, 0.1 mM EDTA, 50 mM NaCl, 10% glycerol, 1 mM PMSF). After 15 min of incubation on ice, insolubilised material was removed by centrifugation at 4°C. The supernatant was mixed with 10x loading dye (5% (w/v) Coomassie Brilliant Blue G-250, 100 mM Tris pH 7, 500 mM 6-aminocaproic acid) and loaded on 4–10% gradient BN-PAGE gels. In-gel complex I activity was determined by incubating the BN-PAGE gels in 2 mM Tris-HCl pH 7.4, 0.1 mg/ml NADH and 2.5 mg/ml iodonitrotetrazolium chloride. In-gel complex IV activity was determined by incubating the BN-PAGE gels in 0.05mM phosphate buffer pH 7.4, 0.5 mg/ml 3.3′-diamidobenzidine tetrahydrochloride (DAB), 1 mg/ml cytochrome *c*, 0.2 M sucrose and 1 μg/ml catalase. Stainings were carried out at room temperature.

### DNA isolation and qPCR

Genomic DNA was isolated from 4 days AEL larvae with the DNeasy Blood and Tissue Kit (Qiagen), following manufacturer’s instructions. Mitochondrial DNA levels were determined by quantitative real-time PCR (qRT-PCR) on a QuantStudio 6 Flex Real-Time PCR System (Thermo Scientific), using Platinum SYBR Green qPCR supermix-UDG (Thermo Scientific). Reactions were carried out in triplicates in a final volume of 20 μL with 5 ng of DNA and 10 pmol of specific primers (primers are listed in S1 Table).

### RNA isolation, quantitative RT-PCR (qRT-PCR) and Northern blot analysis of Dm mitochondrial RNAs

Total RNA was isolated, using the ToTALLY RNA kit (Thermo Scientific) and quantified with a Qubit fluorometer (Thermo Scientific). Reverse transcription was performed using High Capacity cDNA Reverse Transcription Kit (Thermo Scientific). qRT-PCR was performed on a QuantStudio 6 Flex Real-Time PCR System, using the TaqMan Universal Master Mix II, with UNG and TaqMan assays (Thermo Scientific) or Platinum SYBR Green qPCR supermix-UDG (Life Technologies). TaqMan assays and primers used for qPCR are listed in S1 Table.

For Northern blot analysis 3 μg of total RNA was separated by neutral 10% PAGE for mitochondrial tRNA separation or 1% MOPS-formaldehyde agarose gels for mitochondrial mRNAs. Separated RNAs were transferred to Hybond-N+ membranes (GE Healthcare) and hybridised with either randomly [^32^P]-labelled dsDNA probes, [^32^P]-labelled strand-specific RNA probes or with strand-specific [^32^P]-end labelled oligonucleotide probes. Oligonucleotide used to generate all the probes are listed in S1 Table.

### *In organello* transcription and translation assays

Mitochondria were isolated from 4 days AEL larvae and *in organello* transcription assays were performed as previously described [32]. In brief, 200μg of fresh mitochondria were incubated for 45 min in transcription buffer (30μCi [^32^P]-UTP, 25mM sucrose, 75mM sorbitol, 100mM KCl, 10mM K2HPO4, 50μM EDTA, 5mM MgCl2, 1mM ADP, 10mM glutamate, 2.5mM malate, 10mM Tris-HCl pH 7.4 and 5% (w/v) BSA), followed by RNA extraction, separation on a 1% MOPS-formaldehyde agarose gel and transferring to Hybond-N+ membranes (GE Healthcare). Mitochondrial *de novo* translation in isolated mitochondria was assayed as previously described [32], using easy-tag EXPRESS ^35^S protein labelling mix (Perkin Elmer). Equal amounts of mitochondrial protein were separated on 17% SDS-PAGE gels, followed by staining with 1g/L Coomassie Brilliant Blue in 20% ethanol and 10% acetic acid. Gels were then destained, dried and exposed to a PhosphorImager screen to visualise the mitochondrial translation products.

### 3′ RACE (Rapid Amplification of cDNA Ends)

The assay was performed as previously described [71]. In summary, 3 μg of isolated RNA was ligated to a phosphorylated oligonucleotide linker using T4 RNA ligase 1 (New England Biolabs). RNA was precipitated and cDNA synthesis was performed using a primer complementary to the linker sequence (anti-linker) and SuperScript II Reverse Transcriptase (Thermo Scientific). The 3′ end of mitochondrial RNAs was PCR amplified using the anti-linker and gene-specific primers. The PCR products were then cloned into pCRII-TOPO and transformed in One Shot TOP10 *E*. *coli* (Thermo Scientific) according to manufacturer’s instructions. The plasmids were purified and the insert was sequenced using M13 sequencing primers. The linker and primers for the 3′RACE experiments are listed in S1 Table.

### Western blot analysis

Western blot analyses were performed using mitochondrial protein extracts according to the Cell Signaling Technology protocol (CellSignaling). Protein extracts were separated on 4–12% or 12% NUPAGE acrylamide gels (Thermo Scientific) and after transfer to PVDF membranes (Millipore) decorated with the following antibodies: Complex I-subunit NDUFS3 (Abcam ab14711, dilution 1:1000), complex IV-subunit COX3 (Abcam ab110259, 1:500), Tubulin (Sigma, T6199, dilution 1:2000), Histone H3 (Sigma, H0164, dilution 1:1000), Flag (Sigma, F3165, dilution 1:1000) and VDAC1 (Abcam ab14734, dilution 1:1000–2000). Protein bands were visualised with Clarity western ECL substrate (Bio-Rad).

### Immunofluorescence of larvae brains

For J2 immunofluorescence on larvae brain, tissues were dissected in PBS, fixed for 5 minutes in 4% formaldehyde, and washed for 5 minutes. Larvae brain were then permeabilised for 2 hours in 0.5% Triton X-100 in PBS, and saturated for 1 hour in 0.5% BSA, 0.1% Tween 20 in PBS (PBTB). Primary antibodies anti-dsRNA (Scicons J2: anti-dsRNA/100105500) and anti ATP synthase subunit 5a (Abcam ab151229) were used at 1:200 in PBTB for overnight at 4°C, and later washed for 1 hour in 0.1% Tween in PBS (PBTW). Secondary antibodies (Molecular Probes, IgG,568, A-11031, and Life Technologies, Alexa Fluor488, A-11008) were used at 1:500 for 2 hours and washed for 1 hour in PBTW. Preparations were mounted in Vectashield/DAPI (Vector). A LSM880 Zeiss confocal microscope was used for imaging.

Mitochondria of the ventral nerve cord were visualised by crossing *dmpnpase*^*KO*^, *dmsuv3*^*KD*^, *dmlrpprc1*^*KD*,^ *dmmtpap*^*KO*^, or *dmpnpase*^*OE*^*/dmsuv3*^*OE*^ flies to previously generated UAS-mit::dendra2 flies (w;elav-gal4,*uasmit*::*dendra2*;) [54]. For in situ detection of mit::dendra2, living larval central nervous systems were rapidly dissected, mounted into PBS, and immediately imaged, using a LSM880 Zeiss confocal microscope.

### Massive parallel sequencing and computational analysis

Human PNPase (PNPT1; NM_033109) or yeast DSS1p (UniProtKB—P39112) were used in protein BLAST searches against the Dm reference protein database. ClustalW alignment was performed using Geneious R6 software (Biomatters; http://www.geneious.com).

DsRNA-seq was performed as described previously [53] with slight modifications. 5μg of RNA from purified mitochondrial and cytoplasmic fractions were used for IP with anti-dsRNA J2 ab. The RNA was diluted in 1.5ml NET-2 buffer (reconstituted to 10mM MgCl2 and 0.1% NP-40) and incubated with 5μg of J2 ab bound to 50μl Protein-G beads for 2 hrs in cold room. Washings were done twice with HSWB and NET-2 buffer respectively. The bound RNA was extracted using Trizol. The resulting 100 ng of J2-IPed dsRNA were used to make the libraries according to the manual of NEBNext Ultra II Directional RNA Library Prep kit for Illumina (New England Biolabs). Libraries were quantified using Agilent 2100 Bioanalyzer (Agilent Technologies). Libraries were sequenced on Illumina NextSeq 550 with 42bp paired-end reads.

Transcripts per million (TPM) values were determined by quasi-mapping of paired-end reads with salmon v0.11.3 [72] against a cDNA library of BDGP6 (release 94). Normalisation factors were calculated from the average TPM values of all transcripts with average TPM values above 14, which were 28SrRNA-Psi:CR40596, 18SrRNA-Psi:CR41602, 28SrRNA-Psi:45851, 5.8SrRNA-Psi:CR45854, 28SrRNA-Psi:CR45855, 28SrRNA-Psi:CR45859, 18SrRNA-Psi:CR45861 and 28SrRNA-Psi:CR45862, excluding mitochondrial lrRNA and srRNA. Data was visualised with R v3.5.1 (accessed July 2018) (R code Team 2018). IGV views were generated after mapping paired fastq-files with bowtie2 v2.3.4.3 (September 2018, [73]) against a BDGP6 index, and conversion and sorting with samtools v1.9 [74]. Sorted bam-files were indexed and opened with the Integrated Genomics Viewer 2.4.16 Java application [75].

### Statistical analysis

All data were analysed using Prism 6 software and are represented as mean ± standard error of the mean (SEM). An unpaired t-test was used to analyse the statistical significance of the results. The exception is for the poly(A) tail data, where the error bars represent the mean ± SD and Mann-Whitney test was used to analyse the statistical significance of each experimental group sets.

## Supporting information

S1 Fig Graphic representation of the genomic locus of CG11337. **Related to** Fig 1. Protein domains and electropherogram of control (*dmpnpase*) and CRISPR/Cas9 gene edited knockout (*dmpnpase*^*KO*^) samples are shown. Guide RNA is shown in insert.
(TIF)

S2 Fig Genetic and functional characterisation of *dmpnpase*^*KO*^ and *dmpnpase*^*KD*^ samples. **Related to** Figs 1 and 2. (A) *De novo* mitochondrial translation in isolated mitochondria from *dmpnpase*^*KO*^ and control larvae (w;;) at 4 days AEL. Samples were loaded according to protein quantification and controlled by Coomasie Blue staining.(B) Body size comparison in controls (w;;), *dmpnpase*^*KD*^ and *dmpnpase*^*KO*^ larvae at 4 days AEL, scale bar size 1mm. (C) qRT-PCR of *dmpnpase* transcript levels in silenced and controls (as described in B) larvea at 4 days AEL. Ribosomal Protein (RP) 49 transcript was used as an endogenous control. (D) Hatching rates of *dmpnpase*^*KD*^ and control flies as described in B. (E) Isolated respiratory chain enzyme activities in *dmpnpase*^*KD*^ (w;UAS-*dmpnpase*RNAi/+;daGAL4/+) and controls (control 1: w;;daGAL4/+, control 2: w;UAS-*dmpnpase*RNAi/+;) larvae at 4 days AEL. Mitochondrial protein extracts from larvae at 4 days AEL were assessed for complex I (NADH coenzyme Q reductase), complex I+III (NADH–cytochrome *c* reductase), complex II (NADH cytochrome *c* reductase), complex III (succinate dehydrogenase), complex II+III (succinate cytochrome *c* reductase) and complex IV (cytochrome *c* oxidase). (F) *De novo* mitochondrial transcription in isolated mitochondria from *dmpnpase*^*KD*^ (w;UAS-*dmpnpase*RNAi/+;daGAL4/+) and controls (control 1: w;;daGAL4/+, control 2: w;UAS-*dmpnpase*RNAi/+;) larvae at 4 days AEL. Mitochondrial rRNA 12S was used as RNA loading control and porin as a mitochondria input control. (G) Mitochondrial mRNA steady-state levels in *dmpnpase*^*KD*^, and its controls at 4 days AEL by qRT-PCR. RP49 transcript was used as an endogenous control.
(TIF)

S3 Fig Characterisation of mitochondrial RNAs in *dmpnpase*^*KO*^ samples. **Related to** Fig 2. (A) Northern blot analysis by formaldehyde-agarose gel electrophoresis with total RNA isolated from controls (control 1: w;;, control 2: w;;*dmpnpase*^*KO*^/TM6B) and *dmpnpase*^*KO*^ (w;;*dmpnpase*^*KO*^/*dmpnpase*^*KO*^) 4 day AEL larvae. The signals were detected using oligonucleotide probes and single stranded RNA probes. Putative degradation products are indicated by an asterisk (*). Nuclear encoded RP49 was used as a loading control. (B) Northern blot analysis by neutral polyacrylamide gel electrophoresis of the steady-state levels of mitochondrial tRNAs in *dmpnpase*^*KO*^ and controls (as described in B) larvae at 4 days AEL. (C) Poly(A) tail length in individually sequenced clones after 3′ RACE analysis of ND1 transcripts in *dmpnpase*^*KO*^ (n = 50) and control (w;; n = 24).
(TIF)

S4 Fig Validation and characterisation of double mutants. **Related to** Figs 2 and 3. (A) Body size comparison in controls (w;;), *dmpnpase*^*KD*^/*dmsuv3*^*KD*^ (w;UAS-*dmsuv3*RNAi/UAS-*dmpnpase*RNAi;daGAL4/+) and *dmpnpase*^*O*E^/*dmsuv3*^*OE*^ (w;UAS-*dmsuv3*/+;UAS-*dmpnpase*/daGAL4) larvae at 4 days AEL, scale bar size 1mm. (B) Relative amounts of DmPNPase and DmSUV3 mRNA steady-state levels in control (control:w;UAS-*dmsuv3*RNAi/UAS-*dmpnpase*RNAi;), *dmsuv3KD*, *dmpnpaseKD*, and *dmpnpaseKD*/*dmsuv3KD* larvae 4 day AEL. (C) Relative amounts of DmPNPase and DmSUV3 mRNA steady-state levels in control (control: w;UAS-*dmsuv3*/+;UAS-*dmpnpase*/+), *dmsuv3*^*OE*^, *dmpnpase*^*OE*^, and *dmpnpase*^*OE*^/*dmsuv3*^*OE*^ larvae 4 day AEL. (D) Relative amounts of DmPNPase, DmSUV3 and DmMTPAP mRNA steady-state levels in *dmpnpase*^*OE*^, *dmmtpap*^*KO*^ (*dmmtpap*^*KO*^/Y;;),*dmpnpase*^*OE*^/*dmmtpap*^*KO*^ (*dmmtpap*^*KO*^/Y;;UAS-*dmpnpase*/daGAL4), and control larvae 4 day AEL. (E) Northern blot analysis of the steady-state levels of mitochondrial mRNAs in *dmpnpase*^*OE*^, *dmmtpap*^*KO*^, *dmpnpase*^*OE*^/*dmmtpap*^*KO*^, and control larvae 4 day AEL. (F) Relative amounts of DmPNPase, DmSUV3 and DmLRPPRC mRNA steady-state levels in *dmpnpase*^*KD*^ (w;;UAS-*bsf*RNAi#1/daGAL4), *dmpnpase*^*KD*^, *dmsuv3*^*KD*^, *dmpnpase*^*KD*^/*dmlrpprc1*^*KD*^ (w;UAS-*dmpnpase*RNAi/+;UAS-*bsf*RNAi#1/daGAL4), *dmsuv3*^*KD*^/*dmlrpprc1*^*KD*^ (w;UAS-*dmsuv3*RNAi/+;UAS-*bsf*RNAi#1/daGAL4), and control larvae 4 day AEL.
(TIF)

S5 Fig Characterisation of mitochondrial-derived dsRNAs. **Related to** Fig 5. (A) Western blot analysis of total (T), cytosolic (C) and mitochondrial (M) fractions from control, *dmsuv3*^*KD*^, *dmpnpase*^*KO*^, and *dmmtpap*^*KO*^ protein extracts to measure purity. Antibodies decorating Tubulin and Porin were used as cytosolic and mitochondrial markers, respectively. (B) Scatterblot of normalised transcripts per million (TPM) values of all detected transcripts in two replicates of cytosolic and mitochondrial fractions after J2-enrichment. Mono- and bicistronic mRNA and rRNA transcripts encoded on mitochondrial DNA are highlighted in red. The point of normalisation is indicated as the intersection between the two dotted lines. (C) IGV view of total transcript read counts aligned to the coding region of mitochondrial DNA visualised with Integrated Genomics Viewer. The relative height is normalised to the highest peak in the 16S region.
(TIF)

S6 Fig Confocal microscopy images of the ventral nerve cord of *dmpnpase*^*KO*^, *dmsuv3*^*KD*^, *dmmtpap*^*KO*^, *dmlrpprc1*^*KD*^, and *dmmtpnpase*^*OE*^/*dmsuv3*^*OE*^ larvae. **Related to** Fig 5. Mitochondria (green) were visualised at two magnifications by mitochondria-targeted dendra2 fluorescent protein, expressed from the elav-GAL4 driver (w;elav-gal4,uasmit::dendra2;).
(TIF)

S1 Table Taqman probes and oligonucleotide. (PDF)
