# Supplementary figures and images for: Defects of mitochondrial RNA turnover lead to the accumulation of double-stranded RNA in vivo
Source: PLoS Genet. 2019 Jul 31;15(7):e1008240. doi: 10.1371/journal.pgen.1008240 (PMC6668790; doi:10.1371/journal.pgen.1008240)

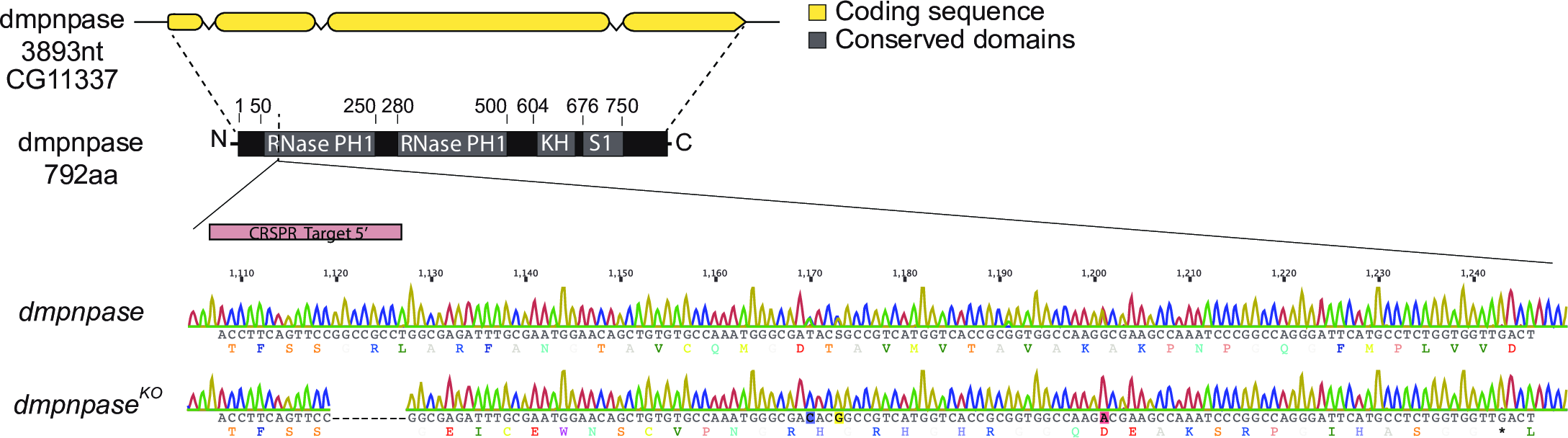

Supplement: S1 Fig — Related to Fig 1. Protein domains and electropherogram of control (dmpnpase) and CRISPR/Cas9 gene edited knockout (dmpnpaseKO) samples are shown. Guide RNA is shown in insert. (TIF) [file pgen.1008240.s001.tif]

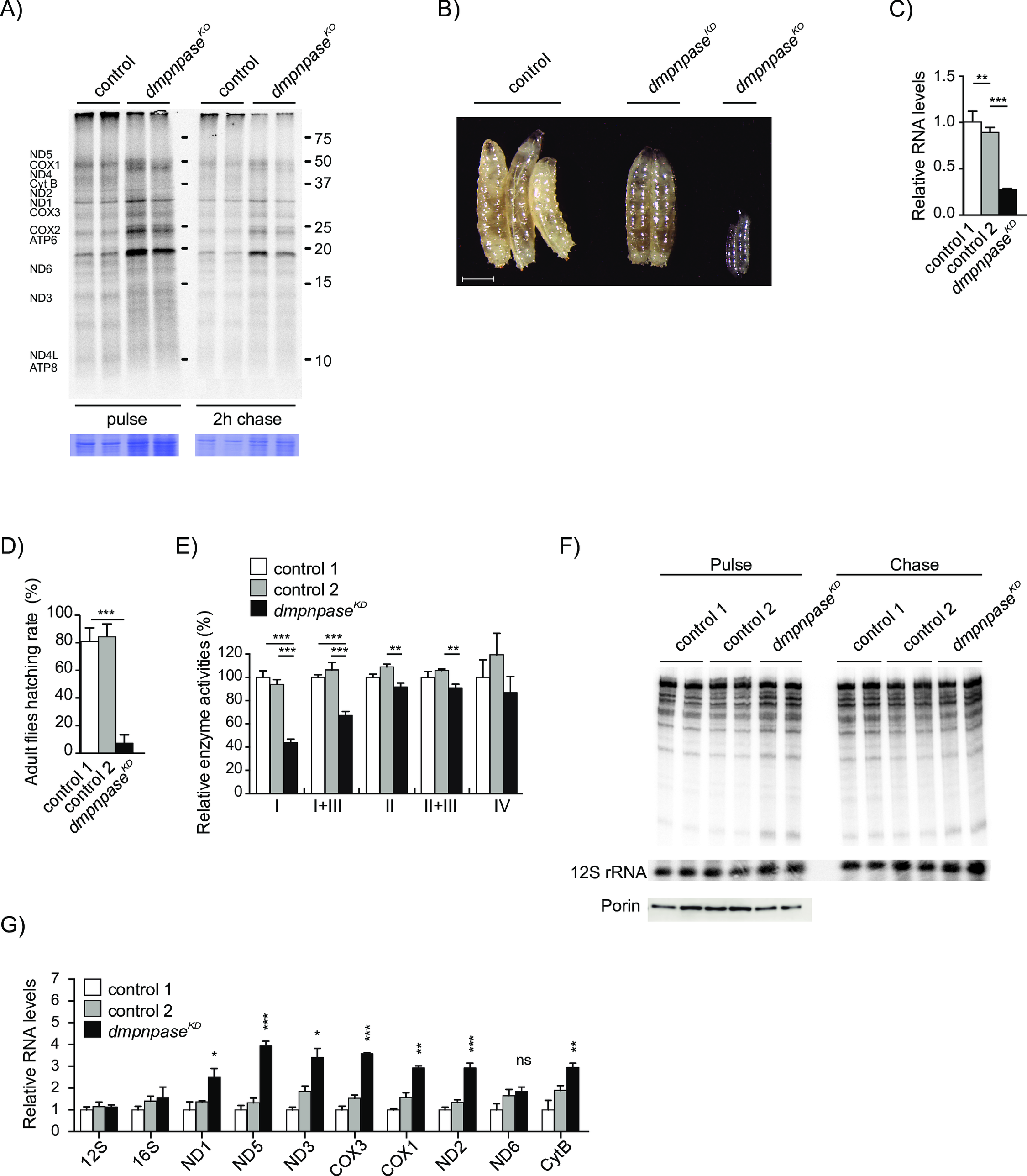

Supplement: S2 Fig — Related to Figs 1 and 2. (A) De novo mitochondrial translation in isolated mitochondria from dmpnpaseKO and control larvae (w;;) at 4 days AEL. Samples were loaded according to protein quantification and controlled by Coomasie Blue staining.(B) Body size comparison in controls (w;;), dmpnpaseKD and dmpnpaseKO larvae at 4 days AEL, scale bar size 1mm. (C) qRT-PCR of dmpnpase transcript levels in silenced and controls (as described in B) larvea at 4 days AEL. Ribosomal Protein (RP) 49 transcript was used as an endogenous control. (D) Hatching rates of dmpnpaseKD and control flies as described in B. (E) Isolated respiratory chain enzyme activities in dmpnpaseKD (w;UAS-dmpnpaseRNAi/+;daGAL4/+) and controls (control 1: w;;daGAL4/+, control 2: w;UAS-dmpnpaseRNAi/+;) larvae at 4 days AEL. Mitochondrial protein extracts from larvae at 4 days AEL were assessed for complex I (NADH coenzyme Q reductase), complex I+III (NADH–cytochrome c reductase), complex II (NADH cytochrome c reductase), complex III (succinate dehydrogenase), complex II+III (succinate cytochrome c reductase) and complex IV (cytochrome c oxidase). (F) De novo mitochondrial transcription in isolated mitochondria from dmpnpaseKD (w;UAS-dmpnpaseRNAi/+;daGAL4/+) and controls (control 1: w;;daGAL4/+, control 2: w;UAS-dmpnpaseRNAi/+;) larvae at 4 days AEL. Mitochondrial rRNA 12S was used as RNA loading control and porin as a mitochondria input control. (G) Mitochondrial mRNA steady-state levels in dmpnpaseKD, and its controls at 4 days AEL by qRT-PCR. RP49 transcript was used as an endogenous control. (TIF) [file pgen.1008240.s002.tif]

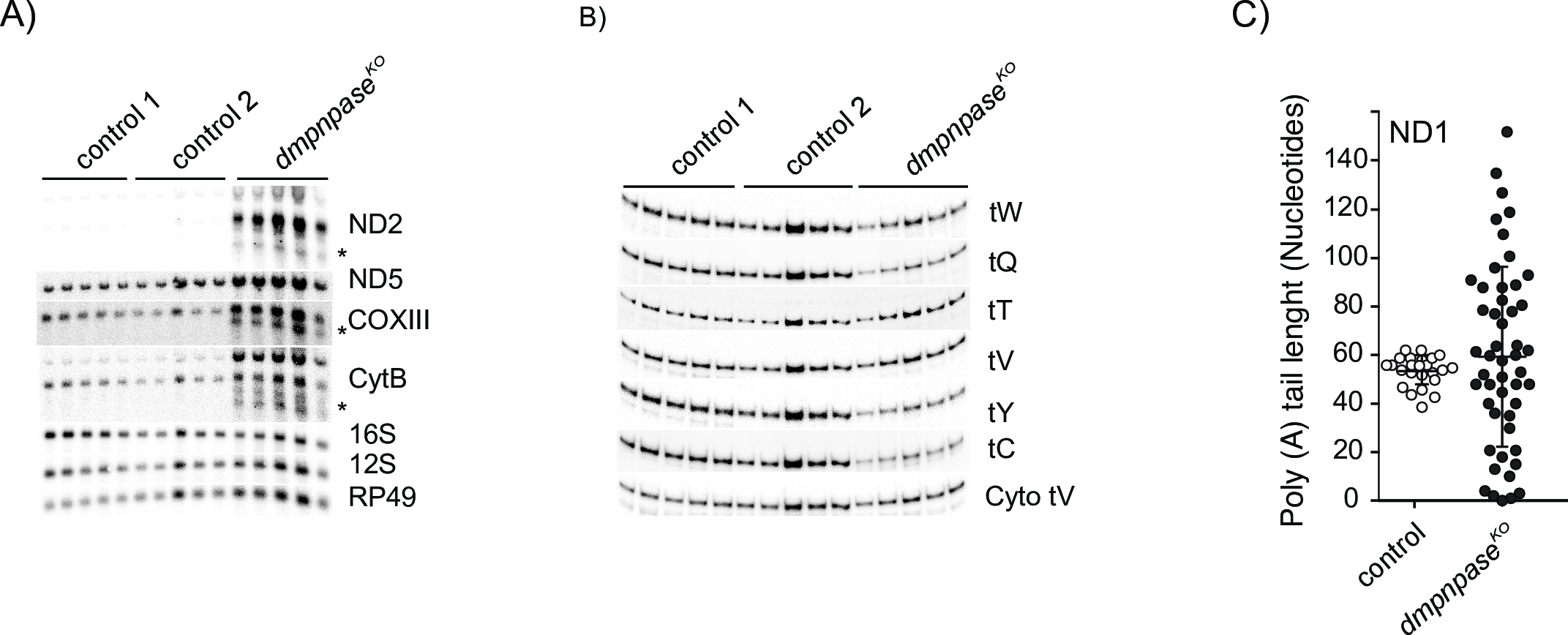

Supplement: S3 Fig — Related to Fig 2. (A) Northern blot analysis by formaldehyde-agarose gel electrophoresis with total RNA isolated from controls (control 1: w;;, control 2: w;;dmpnpaseKO/TM6B) and dmpnpaseKO (w;;dmpnpaseKO/dmpnpaseKO) 4 day AEL larvae. The signals were detected using oligonucleotide probes and single stranded RNA probes. Putative degradation products are indicated by an asterisk (*). Nuclear encoded RP49 was used as a loading control. (B) Northern blot analysis by neutral polyacrylamide gel electrophoresis of the steady-state levels of mitochondrial tRNAs in dmpnpaseKO and controls (as described in B) larvae at 4 days AEL. (C) Poly(A) tail length in individually sequenced clones after 3′ RACE analysis of ND1 transcripts in dmpnpaseKO (n = 50) and control (w;; n = 24). (TIF) [file pgen.1008240.s003.tif]

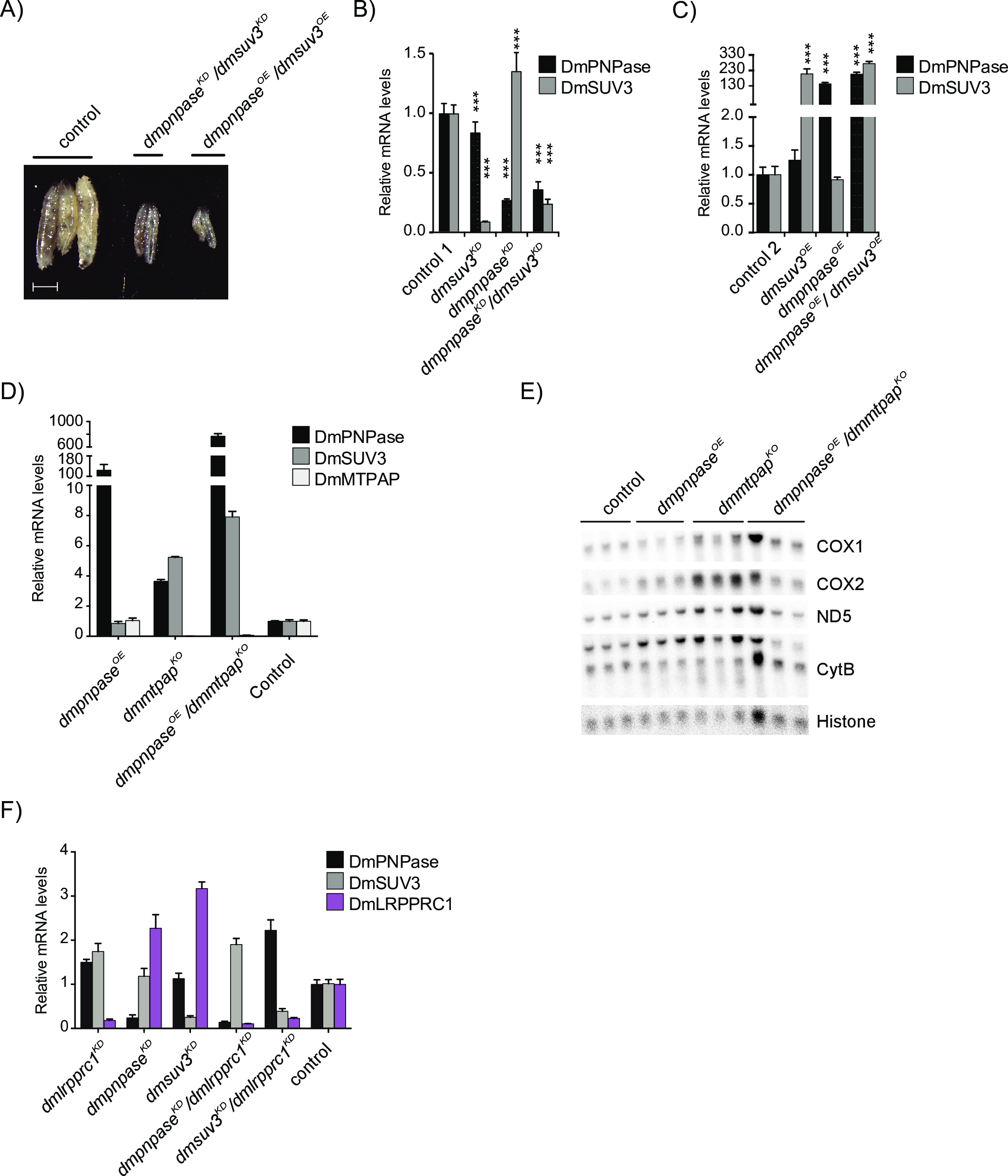

Supplement: S4 Fig — Related to Figs 2 and 3. (A) Body size comparison in controls (w;;), dmpnpaseKD/dmsuv3KD (w;UAS-dmsuv3RNAi/UAS-dmpnpaseRNAi;daGAL4/+) and dmpnpaseOE/dmsuv3OE (w;UAS-dmsuv3/+;UAS-dmpnpase/daGAL4) larvae at 4 days AEL, scale bar size 1mm. (B) Relative amounts of DmPNPase and DmSUV3 mRNA steady-state levels in control (control:w;UAS-dmsuv3RNAi/UAS-dmpnpaseRNAi;), dmsuv3KD, dmpnpaseKD, and dmpnpaseKD/dmsuv3KD larvae 4 day AEL. (C) Relative amounts of DmPNPase and DmSUV3 mRNA steady-state levels in control (control: w;UAS-dmsuv3/+;UAS-dmpnpase/+), dmsuv3OE, dmpnpaseOE, and dmpnpaseOE/dmsuv3OE larvae 4 day AEL. (D) Relative amounts of DmPNPase, DmSUV3 and DmMTPAP mRNA steady-state levels in dmpnpaseOE, dmmtpapKO (dmmtpapKO/Y;;),dmpnpaseOE/dmmtpapKO (dmmtpapKO/Y;;UAS-dmpnpase/daGAL4), and control larvae 4 day AEL. (E) Northern blot analysis of the steady-state levels of mitochondrial mRNAs in dmpnpaseOE, dmmtpapKO, dmpnpaseOE/dmmtpapKO, and control larvae 4 day AEL. (F) Relative amounts of DmPNPase, DmSUV3 and DmLRPPRC mRNA steady-state levels in dmpnpaseKD (w;;UAS-bsfRNAi#1/daGAL4), dmpnpaseKD, dmsuv3KD, dmpnpaseKD/dmlrpprc1KD (w;UAS-dmpnpaseRNAi/+;UAS-bsfRNAi#1/daGAL4), dmsuv3KD/dmlrpprc1KD (w;UAS-dmsuv3RNAi/+;UAS-bsfRNAi#1/daGAL4), and control larvae 4 day AEL. (TIF) [file pgen.1008240.s004.tif]

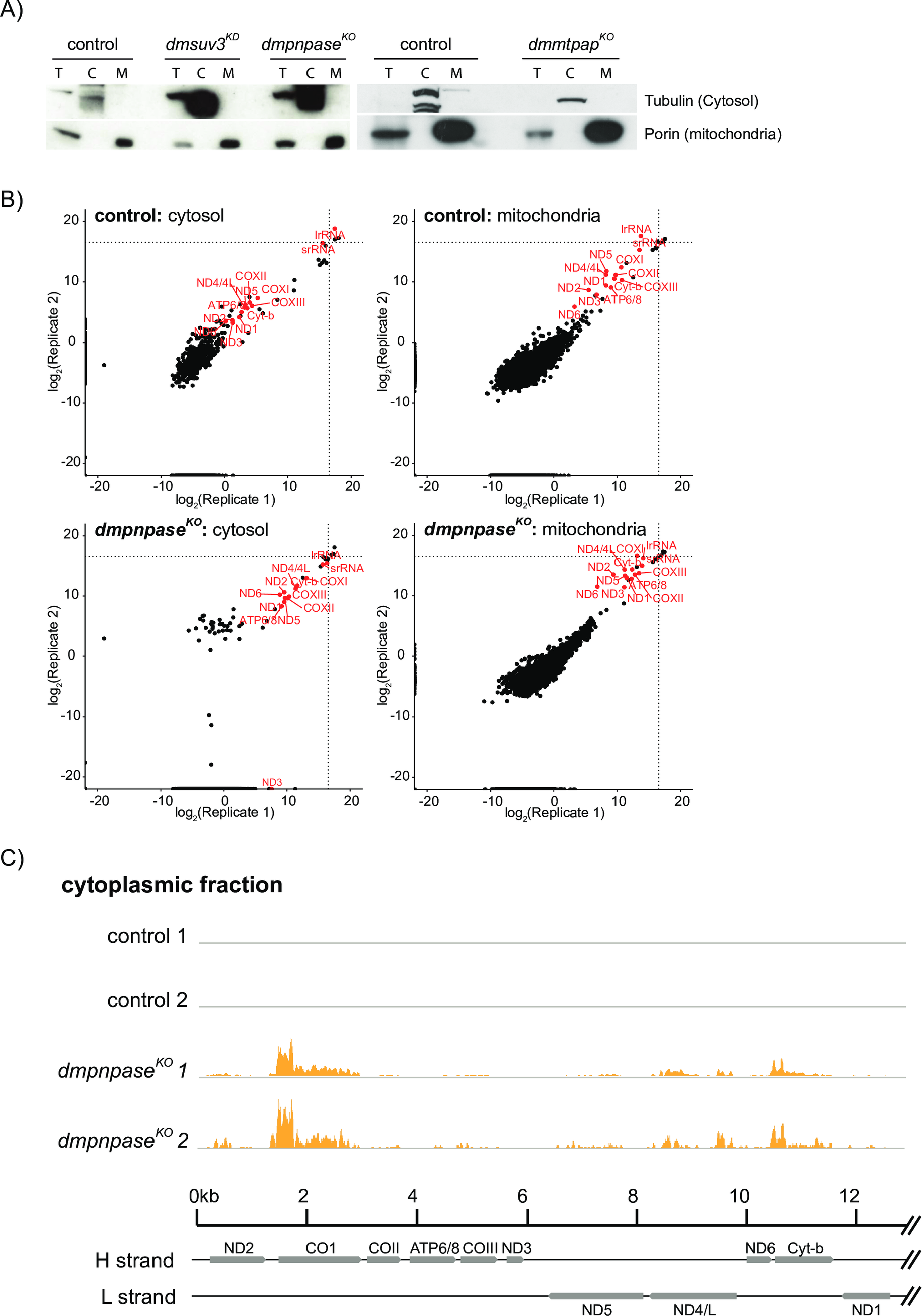

Supplement: S5 Fig — Related to Fig 5. (A) Western blot analysis of total (T), cytosolic (C) and mitochondrial (M) fractions from control, dmsuv3KD, dmpnpaseKO, and dmmtpapKO protein extracts to measure purity. Antibodies decorating Tubulin and Porin were used as cytosolic and mitochondrial markers, respectively. (B) Scatterblot of normalised transcripts per million (TPM) values of all detected transcripts in two replicates of cytosolic and mitochondrial fractions after J2-enrichment. Mono- and bicistronic mRNA and rRNA transcripts encoded on mitochondrial DNA are highlighted in red. The point of normalisation is indicated as the intersection between the two dotted lines. (C) IGV view of total transcript read counts aligned to the coding region of mitochondrial DNA visualised with Integrated Genomics Viewer. The relative height is normalised to the highest peak in the 16S region. (TIF) [file pgen.1008240.s005.tif]

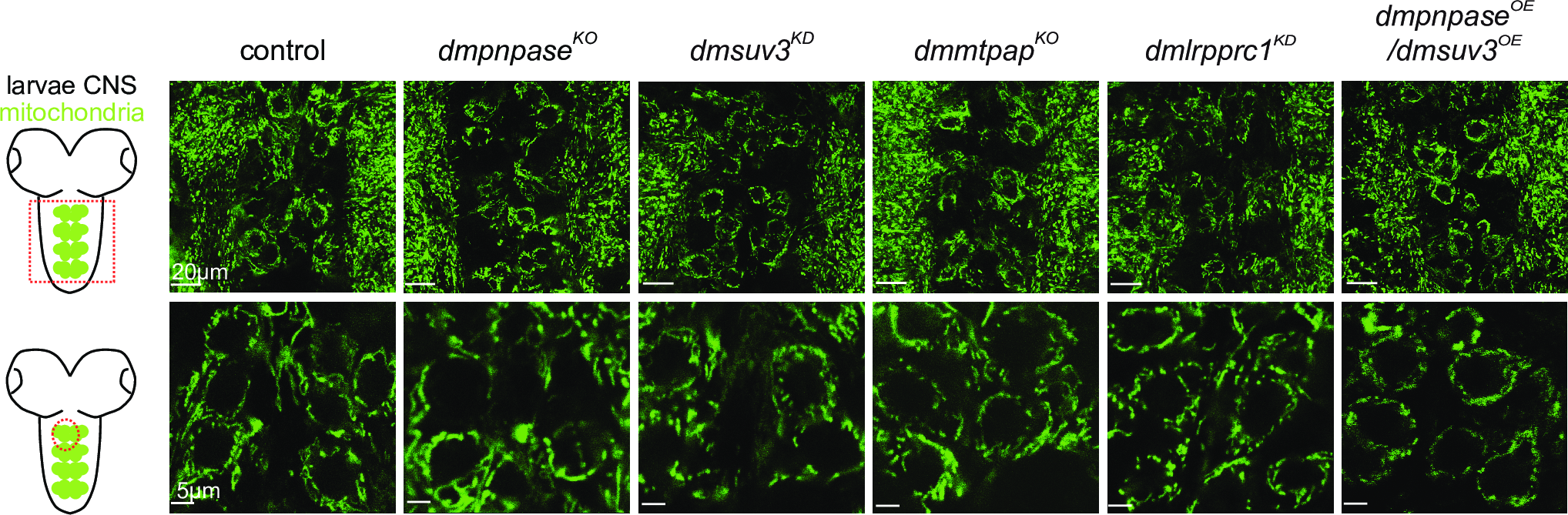

Supplement: S6 Fig — Related to Fig 5. Mitochondria (green) were visualised at two magnifications by mitochondria-targeted dendra2 fluorescent protein, expressed from the elav-GAL4 driver (w;elav-gal4,uasmit::dendra2;). (TIF) [file pgen.1008240.s006.tif]
