# Supplementary material for: Defects of mitochondrial RNA turnover lead to the accumulation of double-stranded RNA in vivo
Source: PLoS Genet. 2019 Jul 31;15(7):e1008240. doi: 10.1371/journal.pgen.1008240 (PMC6668790; doi:10.1371/journal.pgen.1008240)
Supplement: S1 Table — (PDF) [file pgen.1008240.s007.pdf]

S1 Table. Taqman Probes and oligonucleotides used in this study

**Taqman Assays**	
DmMTPNPase	Dm02146799_g1
DmSUV3	Dm02136650_g1
DmRpL32	Dm02151827_g1
DmMT-ND1	AI70K4C
DmMT-ND2	AI20SFG
DmMT-CYTB	AILJJEB
DmMT-ND5	AIHSOVN
DmMT-COX1	AII1M1V
DmMT-COX3	AI89JAK
DmMT-ND3	AIFASI7
DmMT-ND4L	AI5IORW
DmMT-ND6	AI6RMX4
**Primers for cloning PNP-GFP and PNP-FLAG constructs 5'- 3'**	
DmPNPase_XhaI_F	ATGGCCATGATTTTCACAC
DmPNPase_FLAG_XbaI_R	CTCTCCTCTAGACTACTTATCGTCGTCATCCTTGTAATCCGT TCCGCTCTCTCCGGCAG
DmPNPase_Kpn1_R	ATGGTACCCGTTCCGCTCTCTCC
**Primers for generating gRNA and screening for PNPaseKO lines**	
pCFD4_PNP-1_F	TATATAGGAAAGATATCCGGGTGAACTTCGACCTTCAGTTCC GGCCGCCGTTTTAGAGCTAGAAATAGCAAG
pCFD4_PNP-2_R	ATTTTAACTTGCTATTTCTAGCTCTAAAACAATCTAATATTCT GGACATCGACGTTAAATTGAAAATAGGTC
MTPNP_927_ScrPCR1_F	AGCTGCTCAACTATCGCCTC
MTPNP_ScrPCR1_R	CTGGCCGGGATTTGGCTTCG
MTPNPKO_ScrPCR2_F	GACCTTCAGTTCCGGCGA
**Primers for qRT-PCR quantification of mRNAs**	
DmBSF_3809_F	GAGGGATTGATATTGCGGCA
DmBSF_3680_R	CTTGACCACAGAAATCGAT
His2B_F	CTGGCAAGGCTCAGAAGAAC
His2B_R	AAATTCCGGTGTCAGGATGG
12S_F	GATAACGACGGTATATAAACTGATTACA
12S_R	GAGGAACCTGTTTTTTAATCGA
16S_F	ACCTGGCTTACACCGGTTT
16S_R	GGGTGTAGCCGTTCAAATTT
**Primers for qPCR quantification of mtDNA**	
12S_F	GATAACGACGGTATATAAACTGATTACA
12S_R	GAGGAACCTGTTTTTTAATCGA
COX3_F	CAGACTCAATTTATGGATCAACATT
COX3_R	AAAGTTGTTCCGATTAATACATGAA
COX1_F	CAGGATGAACTGTTTATCCACCTTT
COX1_R	AATCCCTGCTAAATGTAGAGAAAAAATAG

ND1_F	AGCCAAACCCCCTCTTCTA
ND1_R	TTTTGATTTTGCTGAAGGAGAAT
RP49_F	CGGATCGATATGCTAAGCTGT
RP49_R	CGACGCACTCTGTTGTCG
**Primers for 3'RACE**	
Linker	ATGTGAGATCATGCACAGTCATA
Anti-linker	GACTGTGCATGATCTCAC
COX1_F	GCTTACACAACATGAAATATTGTATCA
ND1_F (R2)	GGAACTTTACCTCGATTT
16S_F (R2)	GACCTCGATGTTGGATTAAGATATAAT
**Northern blot oligonucleotide probes and primers for double stranded probes**	
tRNA-Gln	AACTTTTGTGCATCATACACCAA
tRNA-Trp	CTTTATTTATAGCTTTGAAGGTTATTAG
tRNA-Cys	GCCTTAGTAAAACTTACTCCTTCAAAA
tRNA-Tyr	ATCTATCGCCTAAACTTCAGCC
tRNA-Gly	AATAGACCTTATGATTGGAAGTCAA
tRNA-Ala	AATGTTATATTTAACTACAACCCT
tRNA-Asn	TCATTAACAGTGATATGCCTC
tRNA-Phe	CTTCAATGTCAAACTCTAGT
tRNA-Thr	TTTTTGATTTACAAGACCAATGTTTT
tRNA-Pro	AGAAATTTCTTTTTCATTAATCCCCAAA
tRNA-Val	TTTGCACAAAAATCTTTTCAATG
tRNA-Met	TGGGGTATGAACCCAGTAG
tRNA-Ser1	CAAAACATATGCTTATTCAAGCTCA
tRNA-Leu2	TTATTGCACTAATCTGCCAAAATAGT
anti-tRNA-Cys	TTTTGAAGGAGTAAGTTTTACTAAGGC
Cytosolic tRNA-Val	GTGATAACCGCTACACTACGGAAA
